# Tuning the Glass Transition Temperature of a Core-Forming Block during Polymerization-Induced Self-Assembly: Statistical Copolymerization of Lauryl Methacrylate with Methyl Methacrylate Provides Access to Spheres, Worms, and Vesicles

**DOI:** 10.1021/acs.macromol.2c00475

**Published:** 2022-05-11

**Authors:** Csilla György, Thomas J. Neal, Timothy Smith, David J. Growney, Steven P. Armes

**Affiliations:** † Dainton Building, Department of Chemistry, University of Sheffield, Brook Hill, Sheffield, South Yorkshire S3 7HF, U.K.; ‡ Lubrizol Ltd., Nether Lane, Hazelwood, Derbyshire DE56 4AN, U.K.

## Abstract

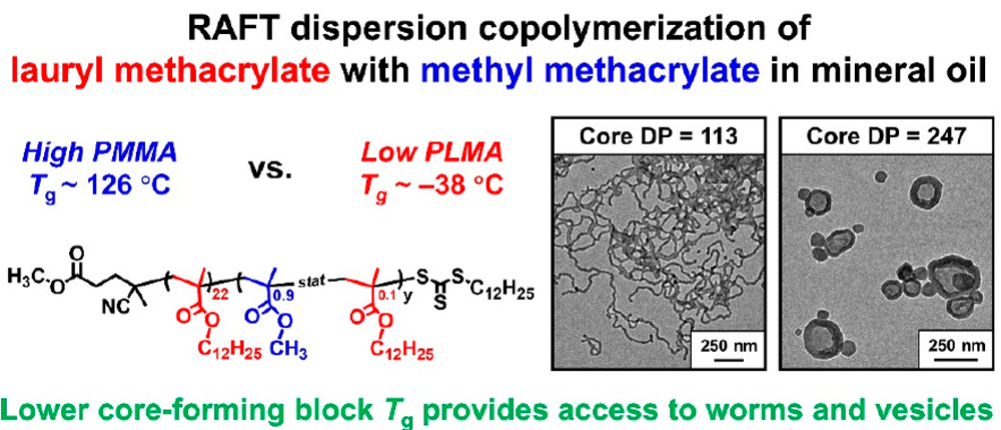

A series of poly(lauryl methacrylate)–poly(methyl methacrylate-*stat*-lauryl methacrylate) (PLMA_*x*_–P(MMA-*stat*-LMA)_*y*_) diblock copolymer nanoparticles were synthesized via RAFT dispersion copolymerization of 90 mol % methyl methacrylate (MMA) with 10 mol % lauryl methacrylate (LMA) in mineral oil by using a poly(lauryl methacrylate) (PLMA) precursor with a mean degree of polymerization (DP) of either 22 or 41. *In situ*^1^H NMR studies of the copolymerization kinetics suggested an overall comonomer conversion of 94% within 2.5 h. GPC analysis confirmed a relatively narrow molecular weight distribution (*M*_w_/*M*_n_ ≤ 1.35) for each diblock copolymer. Recently, we reported an unexpected morphology constraint when targeting PLMA_22_–PMMA_*y*_ nano-objects in mineral oil, with the formation of kinetically trapped spheres being attributed to the relatively high glass transition temperature (*T*_g_) of the PMMA block. Herein we demonstrate that this limitation can be overcome by (i) incorporating 10 mol % LMA into the core-forming block and (ii) performing such syntheses at 115 °C. This new strategy produced well-defined spheres, worms, or vesicles when using the same PLMA_22_ precursor. Introducing the LMA comonomer not only enhances the mobility of the core-forming copolymer chains by increasing their solvent plasticization but also reduces their effective glass transition temperature to well below the reaction temperature. Copolymer morphologies were initially assigned via transmission electron microscopy (TEM) studies and subsequently confirmed via small-angle X-ray scattering analysis. The thermoresponsive behavior of PLMA_22_–P(0.9MMA-*stat*-0.1LMA)_113_ worms and PLMA_22_–P(0.9MMA-*stat*-0.1LMA)_228_ vesicles was also studied by using dynamic light scattering (DLS) and TEM. The former copolymer underwent a worm-to-sphere transition on heating from 20 to 170 °C while a vesicle-to-worm transition was observed for the latter. Such thermal transitions were irreversible at 0.1% w/w solids but proved to be reversible at 20% w/w solids.

## Introduction

Polymerization-induced self-assembly (PISA) is a well-established technique for the rational synthesis of sterically stabilized diblock copolymer nano-objects in the form of concentrated dispersions.^1−9^ Typically, it involves growing an insoluble block from one end of a soluble block in a suitable solvent. Once the former block attains a certain critical degree of polymerization (DP), micellar nucleation occurs to produce nascent nanoparticles, with the latter block acting as a steric stabilizer to prevent macroscopic precipitation. Unreacted monomer diffuses into the nanoparticle cores, which leads to a relatively high local concentration and hence a faster rate of polymerization.^10−15^ This enables very high conversions to be achieved within relatively short time scales, which makes PISA much more efficient than the equivalent solution polymerization.^13,16^ Depending on the monomer solubility within the continuous phase, PISA formulations can be classified as examples of either dispersion (miscible monomer)^4,5,13−15,17−24^ or emulsion (immiscible monomer)^8,25−31^ polymerization.

In principle, the final copolymer morphology (e.g., spheres, worms, or vesicles) simply depends on the relative volume fractions of the soluble and insoluble blocks, as defined by the fractional packing parameter, *P*.^32,33^ Indeed, there are many PISA formulations for which targeting an asymmetric diblock composition (i.e., a relatively long insoluble block) leads to a progressive evolution in copolymer morphology from spheres to worms to vesicles. This is because the DP of the soluble block remains constant, so *P* must gradually increase during the growth of the insoluble block.^3,30^ However, certain synthesis parameters may lead to morphological constraints. For example, it is well-known that kinetically trapped spheres are usually obtained if the steric stabilizer precursor is relatively long^22,34−36^ or has polyelectrolytic character.^37,38^ This is because strong interparticle repulsive forces inhibit sphere–sphere fusion, which is the critical step in the evolution of the copolymer morphology. Another important parameter can be the glass transition temperature (*T*_g_) of the insoluble structure-directing block. For example, we found that the vesicle morphology was inaccessible for a PISA formation involving the alternating copolymerization of styrene with *N*-phenylmaleimide.^39^ In this case, the *T*_g_ of the core-forming block (219 °C)^40^ was well above the reaction temperature (70 °C) so the growing chains were relatively stiff and immobile. This led to the formation of spheres, partially fused worms, and lamellae as the copolymerization progressed. Recently, we reported the synthesis of poly(lauryl methacrylate)–poly(methyl methacrylate) [PLMA–PMMA] diblock copolymer nano-objects via reversible addition–fragmentation chain transfer (RAFT) dispersion polymerization in mineral oil.^15^ For this PISA formulation, only kinetically trapped spheres or relatively short worms could be obtained. This was attributed to the high *T*_g_ of the structure-directing PMMA block (126 °C) relative to the synthesis temperature (70–115 °C). This is actually a rather subtle effect because the *T*_g_ depends on the DP, as indicated by the Flory–Fox equation.^41,42^ Thus, the *T*_g_ is initially low when the insoluble block is relatively short but increases significantly during the polymerization as the PMMA chains grow longer. MMA is the cheapest methacrylic monomer, so using it as a core-forming block in RAFT PISA offers the most cost-effective formulation for the production of diblock copolymer nanoparticles in non-polar media for potential industrial applications. For example, spheres produced in non-polar media have been demonstrated to be effective friction modifiers,^43^ anisotropic worm-like particles can be used as oil thickeners for silicone oils,^12^ and certain types of vesicles can undergo a vesicle-to-worm transition on heating to provide a new high-temperature oil thickening mechanism.^44^

**Scheme 1:**
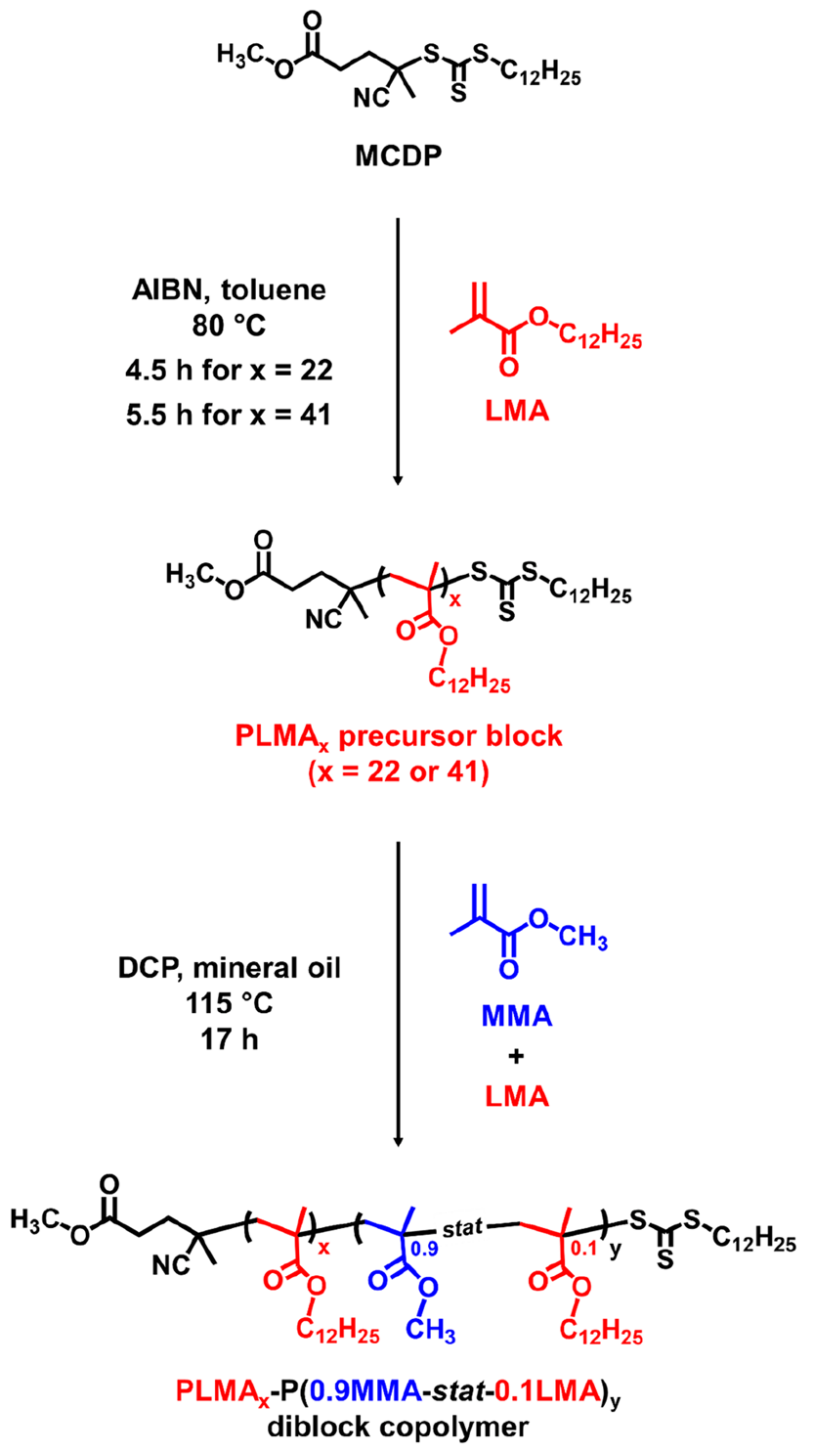
Synthesis of a Poly(lauryl methacrylate) (PLMA_*x*_; *x* = 22 or 41) Precursor via RAFT Solution Polymerization of LMA in Toluene at 50% w/w Solids Using Methyl 4-Cyano-4-(dodecylthiocarbonothioylthio)pentanoate (MCDP) at 80 °C (AIBN Denotes 2,2′-Azoisobutyronitrile), Followed by the RAFT Dispersion Copolymerization of Methyl Methacrylate (MMA) with LMA at 115 °C in Mineral Oil at 20% w/w Solids Using a Dicumyl Peroxide (DCP) Initiator

Herein we report that the PLMA–PMMA PISA formulation can be readily modified to provide convenient access to spheres, worms, or vesicles. This involves conducting a two-pot synthesis protocol whereby lauryl methacrylate is statistically copolymerized with methyl methacrylate during the second-stage polymerization (see Scheme 1). Dispersion polymerization conditions can be maintained if the former comonomer constitutes a minor component (up to ∼20 mol %) relative to the latter. Importantly, this approach is sufficient to lower the effective *T*_g_ of the structure-directing block, which provides convenient access to well-defined spheres, highly anisotropic worms, and vesicles.

## Results and Discussion

### Synthesis of PLMA Precursor Blocks

The PLMA_22_ and PLMA_41_ precursors were synthesized via RAFT solution polymerization of LMA in toluene at 80 °C by using MCDP as a RAFT agent (see Scheme 1). The polymerization was quenched after 4.5 h for PLMA_22_ and after 5.5 h for PLMA_41_ to minimize the loss of RAFT end-groups under monomer-starved conditions.^23^ LMA conversions of 91% (targeting DP = 20) and 89% (targeting DP = 50) were determined by ^1^H NMR analysis. Narrow molecular weight distributions (*M*_w_/*M*_n_ ≤ 1.13) were confirmed by THF GPC analysis, indicating that relatively good RAFT control was obtained during each polymerization.

### Optimizing the Synthesis of PLMA_22_–P(MMA-*stat*-LMA)_*y*_ Diblock Copolymer Nano-Objects

**Figure 1:**
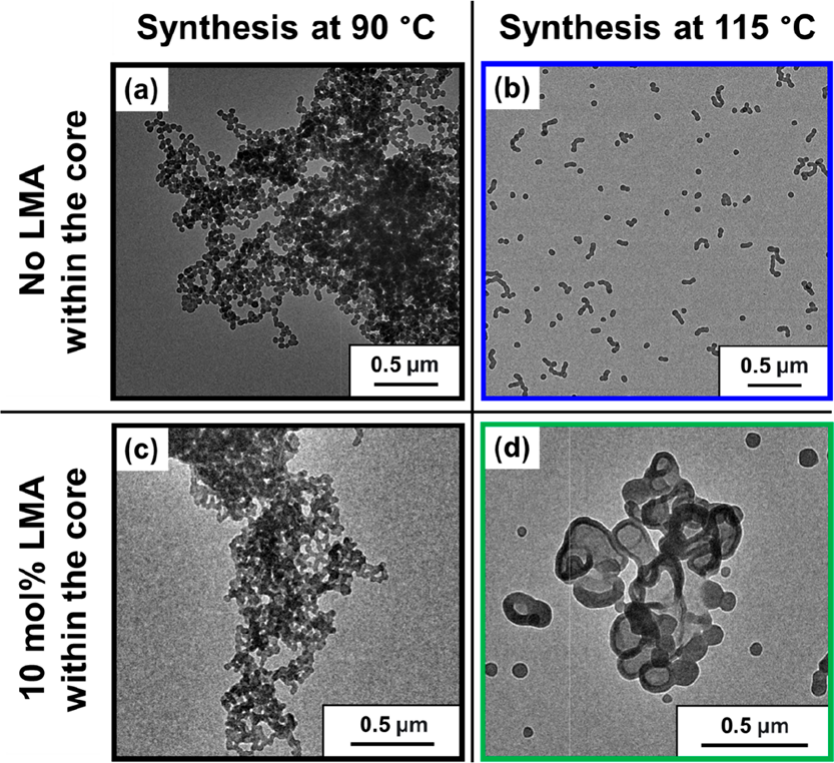
Representative TEM images obtained when targeting (a) PLMA_22_–PMMA_300_ at 90 °C, (b) PLMA_22_–PMMA_300_ at 115 °C, (c) PLMA_22_–P(0.9MMA-*stat*-0.1LMA)_300_ at 90 °C, and (d) PLMA_22_–P(0.9MMA-*stat*-0.1LMA)_300_ at 115 °C using a two-pot synthesis protocol at 20% w/w solids in mineral oil.

When preparing PLMA_22_–PMMA_*y*_ diblock copolymer nano-objects in mineral oil,^15^ we found that only spheres and short worms (*y* < 108) could be accessed even when using a relatively short steric stabilizer block (PLMA_22_). Targeting relatively high DPs (*y* ≥ 108) for the core-forming PMMA block invariably resulted in colloidally unstable micrometer-sized spherical aggregates (see Figure 1a for an example when targeting PLMA_22_–PMMA_300_). The same morphology constraint was also observed when particles were targeted (i) at higher solids, (ii) in an alternative solvent (*n*-dodecane), or (iii) when an alternative poly(stearyl methacrylate) (PSMA_10_) precursor block was utilized. Varying the PISA synthesis conditions did not resolve this problem, thus we hypothesized that it was most likely related to the relatively high *T*_g_ of the core-forming PMMA block.^15^ According to the PISA literature, access to higher order morphologies can be restricted when the synthesis temperature is relatively low compared to the *T*_g_ of the core-forming polymer, which has been explained in terms of insufficient chain mobility.^39,45,46^ In principle, this problem might be eliminated by conducting the PISA synthesis of PLMA_22_–PMMA_*y*_ nanoparticles at a higher temperature.^45,47^ However, only spherical nanoparticles were obtained when targeting PMMA DPs between 50 and 400 at 115 °C, rather than 90 °C (see Figure 1b).^15^

Several research groups have demonstrated that introducing a small amount of a solvophilic comonomer into the core-forming block via statistical copolymerization can greatly influence the nanoparticle morphology obtained during PISA.^46,48−52^ The solvophilic comonomer enhances plasticization of the growing insoluble chains, which results in a higher packing parameter and hence promotes the evolution in morphology from spheres to worms to vesicles. For example, Shi et al. reported the dispersion copolymerization of styrene (St) and 4-vinylpyridine (4VP) using a PEG_45_ precursor block in a methanol/water mixture. In this case, the nanoparticle morphology could be tuned either by varying the DP of the statistical core-forming P(St-*stat*-4VP) block or by adjusting the [St]/[4VP] molar ratio.^49^ Similarly, Zhou et al. studied a poly(2-hydroxyethyl acrylate)–poly(styrene-*stat*-methyl methacrylate) PISA formulation. Only kinetically trapped spheres were obtained when targeting PHEA_21_–PSt_*y*_ diblock copolymer nano-objects (where *y* = 50, 70, or 100) at 20% w/w solids in methanol. In contrast, replacing 25 mol % of the styrene with MMA provided access to spheres, worms, or vesicles when targeting the same overall core-forming block DP.^46^ Figg et al. reported some degree of control over the mean worm length by adjusting the hydrophobic character of the core-forming block. In this aqueous PISA formulation, a poly(*N*,*N*′-dimethylacrylamide) (PDMA) precursor was chain-extended by using both diacetone acrylamide (DAAm) and *N*,*N*′-dimethylacrylamide (DMA).^51^ Similarly, Tan et al. used the same monomer (acrylic acid) to both prepare the stabilizer block and act as a suitable comonomer for the core-forming block to promote the formation of higher order morphologies for poly(acrylic acid)-*block*-poly(acrylic acid-*stat*-styrene) nano-objects in ethanol/water mixtures.^52^

Inspired by these literature examples, we decided to explore replacing a small amount of MMA (ca. 10 mol %) with LMA when generating the core-forming block. TEM images recorded when targeting PLMA_22_–PMMA_300_ nanoparticles at (a) 90 °C and (b) 115 °C are shown in Figure 1. Targeting PLMA_22_–P(0.9MMA-*stat*-0.1LMA)_300_ nano-objects at 90 °C resulted in large, highly polydisperse spherical aggregates (see Figure 1c) with an apparent *z*-average diameter of 1703 nm as judged by DLS, much like that produced during the attempted synthesis of PLMA_22_–PMMA_300_ nanoparticles (*z*-average diameter = 957 nm) at 90 °C. However, when the same PLMA_22_–P(0.9MMA-*stat*-0.1LMA)_300_ composition was targeted at 115 °C (see Figure S1), well-defined vesicles (*z*-average diameter = 148 nm; polydispersity index (PDI) = 0.08) were obtained as a pure phase (see Figure 1d). Thus, it appears that such nano-objects must be prepared at 115 °C to ensure sufficient mobility for the growing diblock copolymer chains to access higher order morphologies.

**Figure 2:**
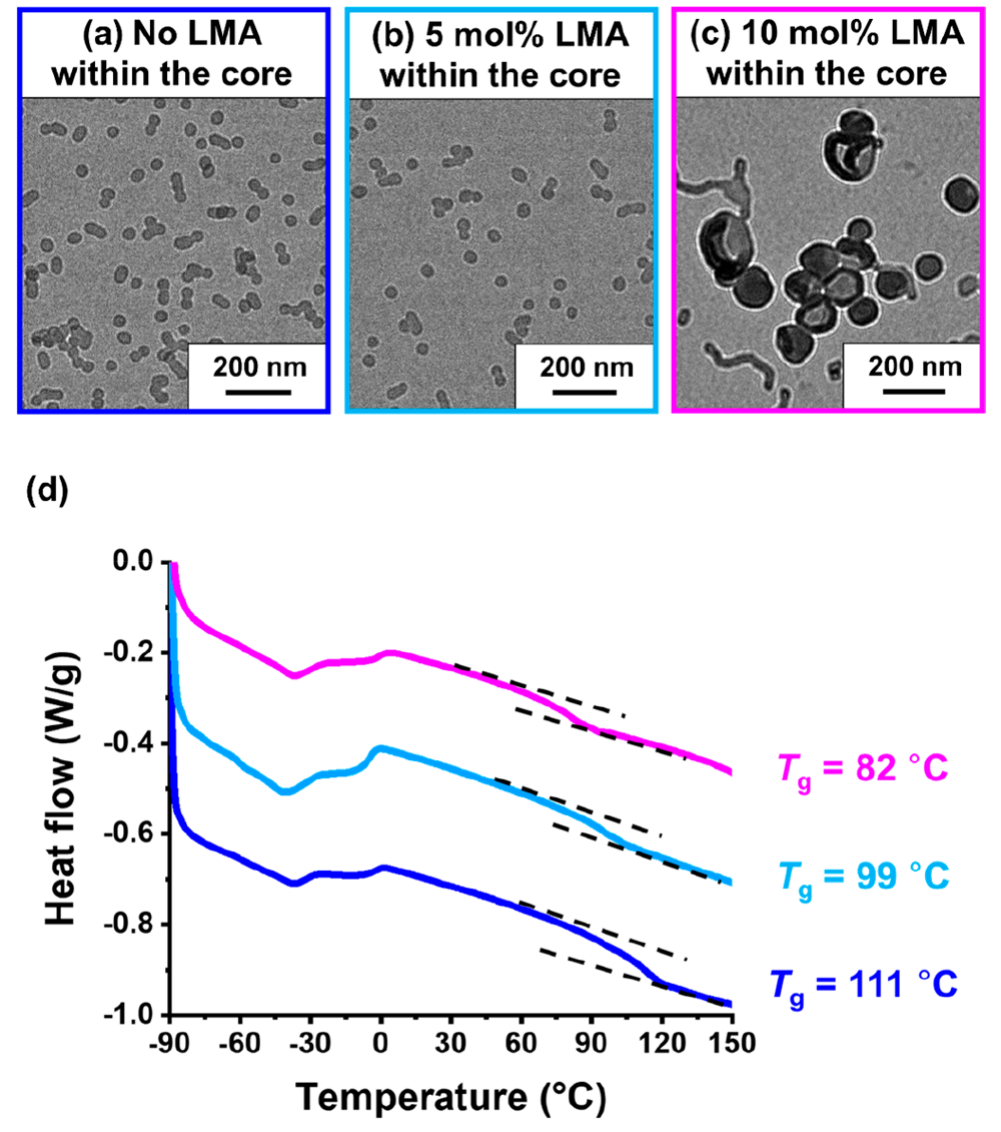
Representative transmission electron microscopy (TEM) images obtained when targeting (a) PLMA_22_–PMMA_200_, (b) PLMA_22_–P(0.95MMA-*stat*-0.05LMA)_200_, or (c) PLMA_22_–P(0.9MMA-*stat*-0.1LMA)_200_ at 20% w/w solids in mineral oil at 115 °C. (d) DSC curves and corresponding calculated core-forming block *T*_g_ values for PLMA_22_–PMMA_192_ (dark blue curve), PLMA_22_–P(0.95MMA-*stat*-0.05LMA)_188_ (light blue curve), and PLMA_22_–P(0.9MMA-*stat*-0.1LMA)_188_ (pink curve). Copolymers were purified by three consecutive precipitations into excess methanol (with redissolution in THF) followed by filtration and drying under vacuum.

Next, we examined the effect of varying the LMA content of the core-forming block (from 0 to 10 mol %) on the copolymer morphology when targeting an overall core-forming DP of 200. Targeting PLMA_22_–PMMA_200_ at 115 °C produced PLMA_22_–PMMA_192_ spheres at 96% conversion (see Figure 2a).^15^ Introducing 5 mol % LMA into the core-forming block was insufficient to produce a higher order morphology, merely resulting in PLMA_22_–P(0.95MMA-*stat*-0.05LMA)_188_ spheres (see Figure 2b**)**. However, employing 10 mol % LMA led to a mixed phase comprising mainly vesicles and worms with a minor population of spheres (see Figure 2c). Clearly, a relatively high synthesis temperature of 115 °C is a necessary but not sufficient condition: incorporation of at least 10 mol % LMA within the core-forming block is also required to access higher order morphologies for the current PISA formulation.

In 2021, we reported that the morphology constraint observed for PLMA_22_–PMMA_*y*_ nano-objects was related to the relatively high *T*_g_ of the core-forming PMMA block.^15^ Flory and Fox derived a well-known equation for calculating the *T*_g_ for statistical copolymers.^41,53^ Because PLMA has a relatively low *T*_g_ of −38 °C (see Figure S2), incorporating LMA comonomer via statistical copolymerization must lead to a reduction in the *T*_g_ of the core-forming block. To examine this hypothesis, three diblock copolymers prepared with a common target core-forming block DP of 200 were purified and subsequently analyzed by DSC. Increasing the LMA content from 0 to 10 mol % led to a gradual *T*_g_ reduction from 111 to 82 °C (see Figure 2d). Thus, introducing LMA comonomer significantly enhances the mobility of the core-forming block at 115 °C by both lowering its *T*_g_ and also increasing its degree of plasticization by hot solvent (mineral oil).

### RAFT Dispersion Copolymerization of MMA with 10 mol % LMA Comonomer at 115 °C

**Figure 3:**
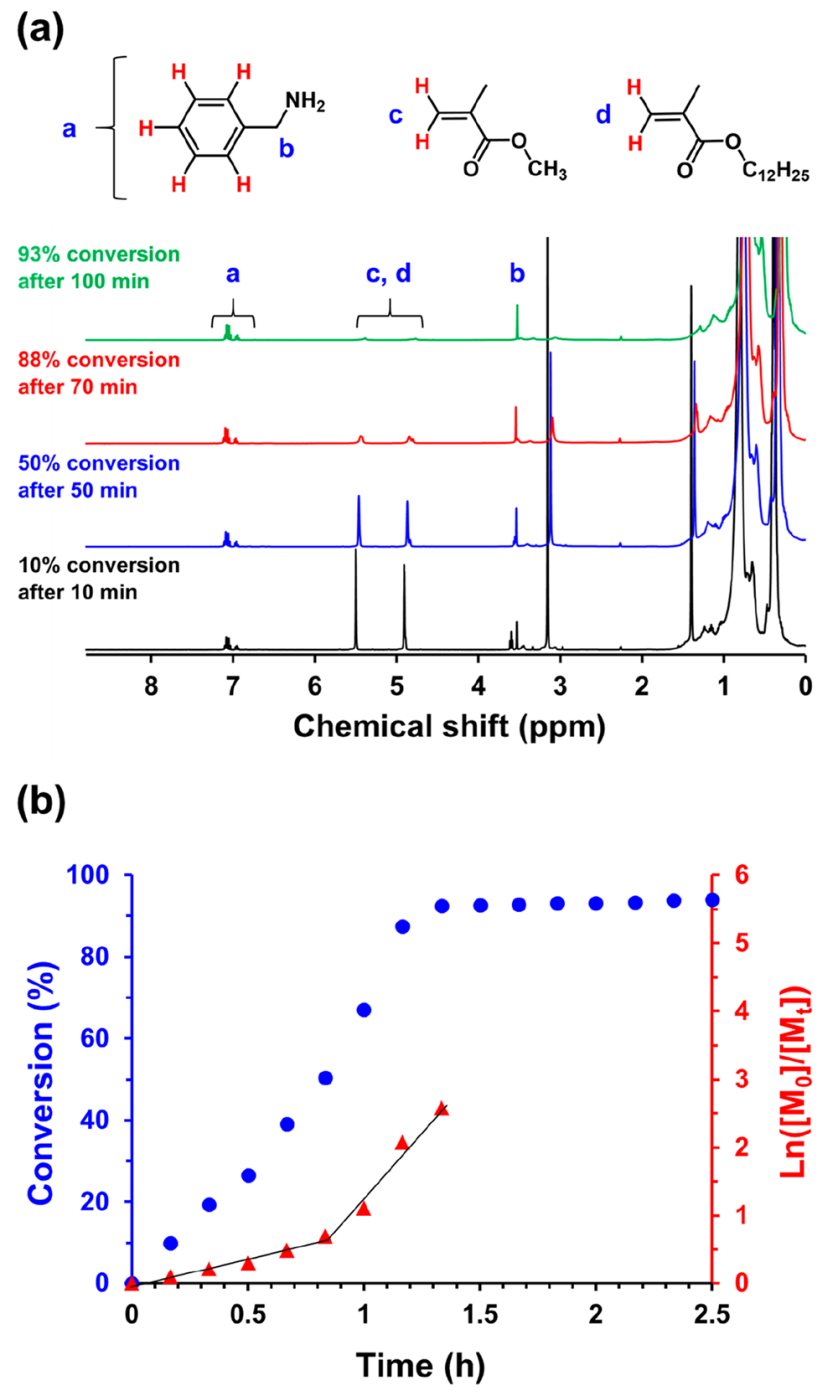
(a) Selected ^1^H NMR spectra recorded during the RAFT dispersion copolymerization of MMA with LMA at 115 °C when targeting PLMA_22_–P(0.9MMA-*stat*-0.1LMA)_300_ vesicles at 20% w/w solids in mineral oil: *t* = 10 min (black spectrum), *t* = 50 min (blue spectrum), *t* = 70 min (red spectrum), and *t* = 100 min (green spectrum) with benzylamine in *d*_6_-DMSO as an external standard. (b) Conversion vs time curve (blue circles) and corresponding ln([M_0_]/[M_*t*_]) vs time plot (red triangles) obtained for the same PISA formulation.

Kinetic studies were performed during the synthesis of PLMA_22_–P(0.9MMA-*stat*-0.1LMA)_282_ vesicles at 20% w/w solids in mineral oil at 115 °C using *in situ*^1^H NMR spectroscopy. Unfortunately, the vinyl signals for the LMA and MMA monomers overlap, so only the overall comonomer conversion could be determined over time. This was achieved by comparing the integrated vinyl signals at 4.5–6.0 ppm to the integrated aromatic signals assigned to an external standard (benzylamine) at 6.5–7.6 ppm (see Figure 3a). Initially, the polymerization proceeded slowly followed by an approximate five-fold rate enhancement after 50 min (see Figure 3b). This time point corresponds to micellar nucleation, whereby the growing second block becomes insoluble in the reaction mixture and the copolymer chains undergo *in situ* self-assembly to form nascent spherical nuclei.^12,35,38^ The instantaneous comonomer conversion was 50% at this point, which corresponds to a mean core-forming block DP of 150. Following nucleation, this statistical copolymerization followed first-order kinetics and attained an overall conversion of 92%. Then a slower rate of polymerization occurred under monomer-starved conditions. A final monomer conversion of 94% was achieved within 2.5 h. THF GPC analysis indicated a relatively narrow molecular weight distribution for the final diblock copolymer (*M*_n_ = 34800 g mol^–1^; *M*_w_/*M*_n_ = 1.25), and the vesicle morphology was confirmed by TEM analysis (see Figure S3). Subsequently, DLS studies indicated a *z*-average diameter of 148 nm (PDI = 0.08).

It is worth asking whether the incomplete monomer conversion (6% unreacted comonomer overall, estimated to be ∼3.6% MMA and ∼2.4% LMA) contributes to the formation of higher order morphologies via plasticization of the statistical core-forming block at 115 °C. In this context, it is notable that targeting PLMA_22_–PMMA_*y*_ (*y* = 50–400) nano-objects at 20% w/w solids in mineral oil invariably resulted in the formation of kinetically trapped spherical nanoparticles despite MMA conversions remaining incomplete (≥95%) after 17 h at 115 °C.^15^ Herein, a 20% w/w dispersion of PLMA_22_–PMMA_291_ spherical nanoparticles (previously prepared at 115 °C in mineral oil; final MMA conversion = 97%) was heated at 115 °C for 17 h in the presence of a large excess amount of LMA monomer ([LMA]/[PLMA_22_–PMMA_291_] molar ratio = 50). The reaction mixture was sealed to prevent evaporative loss of LMA (plus residual MMA comonomer) and was not degassed prior to heating to prevent further copolymerization. The ^1^H NMR spectrum shown in Figure S4 confirms the continued presence of unreacted comonomers after such thermal annealing. Moreover, the GPC trace recorded for the PLMA_22_–PMMA_291_ prior (*M*_n_ = 35000 g mol^–1^; *M*_w_/*M*_n_ = 1.27) was essentially identical with that obtained after this experiment (*M*_n_ = 34900 g mol^–1^; *M*_w_/*M*_n_ = 1.27), confirming that no further copolymerization had occurred. Importantly, TEM images recorded for the PLMA_22_–PMMA_291_ nanoparticles after heating in the presence of LMA indicated the same morphology as the original spheres (see Figure S4). Thus, there is no evidence that LMA monomer can plasticize PMMA cores under such conditions. This is consistent with our observation that LMA is a non-solvent for PMMA homopolymer at 20 °C. In summary, the relatively low residual LMA content that remains after the PISA synthesis of PLMA_22_–P(0.9MMA-*stat*-0.1LMA)_282_ vesicles does not appear to be sufficient to account for the observed evolution in copolymer morphology.

Both Fielding et al.^35,36^ and Derry et al.^11^ reported the RAFT dispersion polymerization of benzyl methacrylate using PLMA precursors in various non-polar solvents. However, Cornel et al. was the first to examine PLMA–PMMA formulations, with such PISA syntheses utilizing a relatively long PLMA_39_ precursor to target spherical nanoparticles in *n*-dodecane.^54^ When revisiting this PLMA–PMMA formulation, PLMA_22_, PLMA_30_, and/or PLMA_41_ precursors were employed to target PMMA DPs of 20–200 at 70, 90, or 115 °C in mineral oil.^15^ In all cases, THF GPC analysis confirmed relatively narrow molecular weight distributions (*M*_w_/*M*_n_ ≤ 1.39), suggesting reasonably good RAFT control.^11,15,35,36,54^ Moreover, we also compared the relative merits of a two-pot synthesis with a one-pot synthesis. A low molecular weight shoulder corresponding to unreacted PLMA precursor^15,35^ became increasingly prominent when targeting higher PMMA DPs using the former protocol. Hence the one-pot protocol always resulted in narrower molecular weight distributions.

In principle, either synthetic route could be employed to produce the PLMA_22_–P(0.9MMA-*stat*-0.1LMA)_*y*_ nanoparticles reported herein. Thus, a two-pot protocol would simply involve the chain extension of a PLMA_22_ precursor via statistical copolymerization of 10 mol % LMA with 90 mol % MMA. This approach has been previously reported by several research groups.^46,49,50^ Alternatively, a one-pot protocol could be utilized in which either MMA or an MMA/LMA mixture was added at a specific (known) LMA conversion. Thus, any unreacted LMA remaining from the first step becomes statistically copolymerized within the insoluble structure-directing block during the subsequent chain extension. This approach was demonstrated for the synthesis of poly(acrylic acid)–poly(acrylic acid-*stat*-styrene) nano-objects by Tan et al.^52^ As indicated in Figure 2, the PLMA_22_–P(MMA-*stat*-LMA)_*y*_ formulation requires incorporation of at least 10 mol % LMA into the insoluble block to access higher order morphologies. Unfortunately, in our hands the kinetics of LMA homopolymerization during the first step was not sufficiently reproducible to ensure precisely the same intermediate LMA conversion after a given reaction time. Rather than produce diblock copolymers with slightly differing stabilizer DPs (and hence introduce corresponding uncertainty regarding the comonomer composition of the insoluble block), we chose to use the two-pot synthesis protocol despite its imperfect blocking efficiency (which is presumably the result of a minor fraction of trithiocarbonate chain-ends being lost during isolation and purification of the PLMA_22_ precursor).

**Figure 4:**
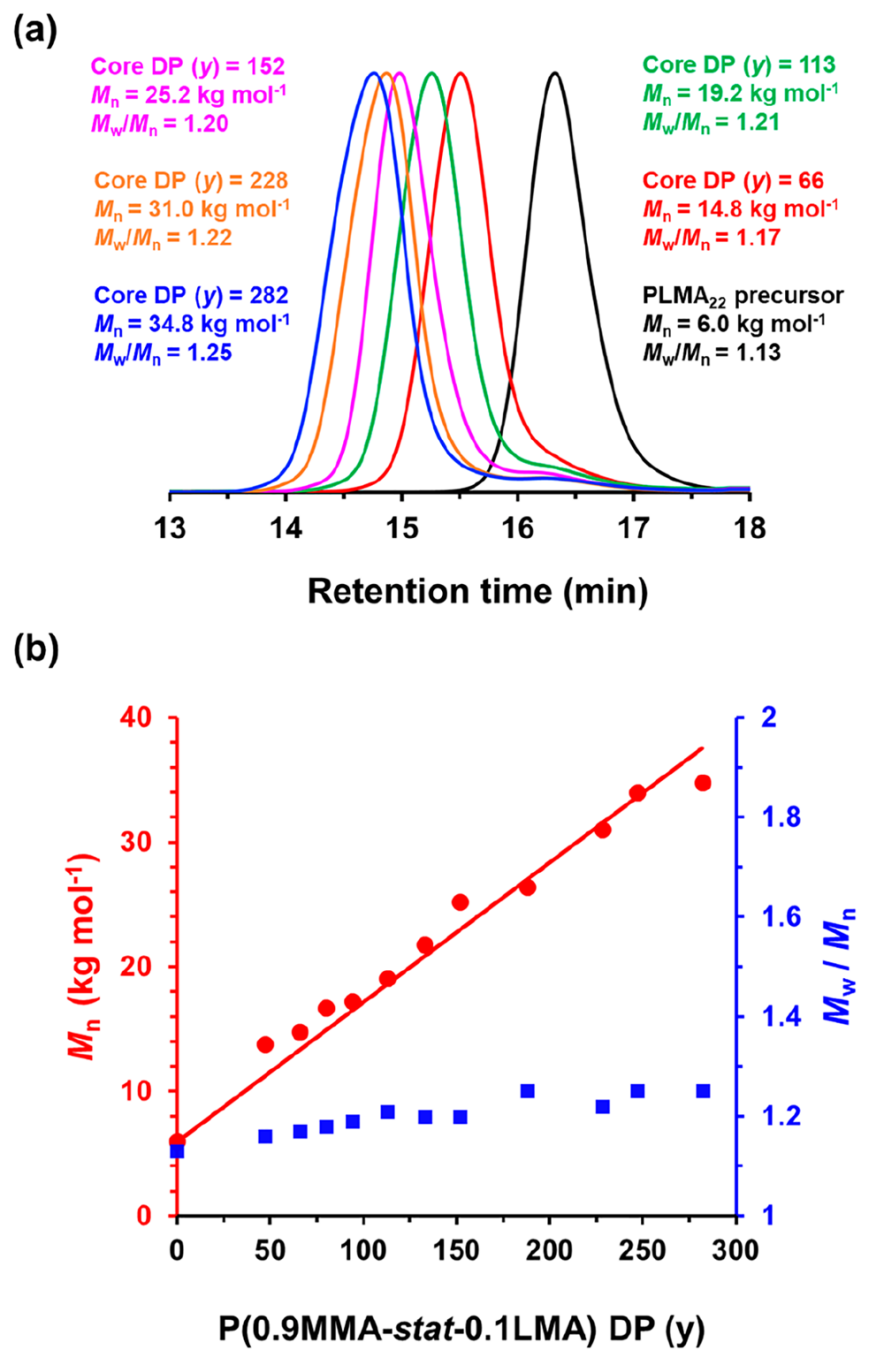
(a) GPC traces (using a series of near-monodisperse poly(methyl methacrylate) calibration standards) recorded using a refractive index detector for a PLMA_22_ precursor (prepared in toluene at 50% w/w solids at 80 °C) and a series of five PLMA_22_–P(0.9MMA-*stat*-0.1LMA)_*y*_ diblock copolymers prepared by RAFT dispersion copolymerization of MMA with LMA comonomer at 115 °C at 20% w/w solids in mineral oil, targeting *y* = 70, 120, 160, 240 or 300. (a) Linear relationship between GPC *M*_n_ (red circles) and P(0.9MMA-*stat*-0.1LMA) DP (as determined by ^1^H NMR studies) for a series of PLMA_22_–P(0.9MMA-*stat*-0.1LMA)_*y*_ diblock copolymers prepared at 20% w/w solids. The corresponding *M*_w_/*M*_n_ (blue squares) data are also shown.

GPC traces recorded for the PLMA_22_ precursor and a series of five PLMA_22_–P(0.9MMA-*stat*-0.1LMA)_*y*_ diblock copolymers prepared using the two-pot protocol are shown in Figure 4a when targeting a core-forming block DP (*y*) of 70, 120, 160, 240, or 300. For each of these copolymers, the overall comonomer conversion is at least 94%. In this case, the low molecular weight shoulder assigned to PLMA precursor is relatively small and narrow molecular weight distributions are obtained (*M*_w_/*M*_n_ ≤ 1.25) (see Table S1). Moreover, a linear correlation between the GPC *M*_n_ and the actual core-forming block DP (corrected for the final comonomer conversion) is evident in Figure 4b. In summary, a two-pot synthesis leads to acceptable results under the stated conditions.

### Construction of a Pseudo-Phase Diagram for a Series of PLMA_*x*_–P(0.9MMA-*stat*-0.1LMA)_*y*_ Diblock Copolymer Nano-Objects

**Figure 5:**
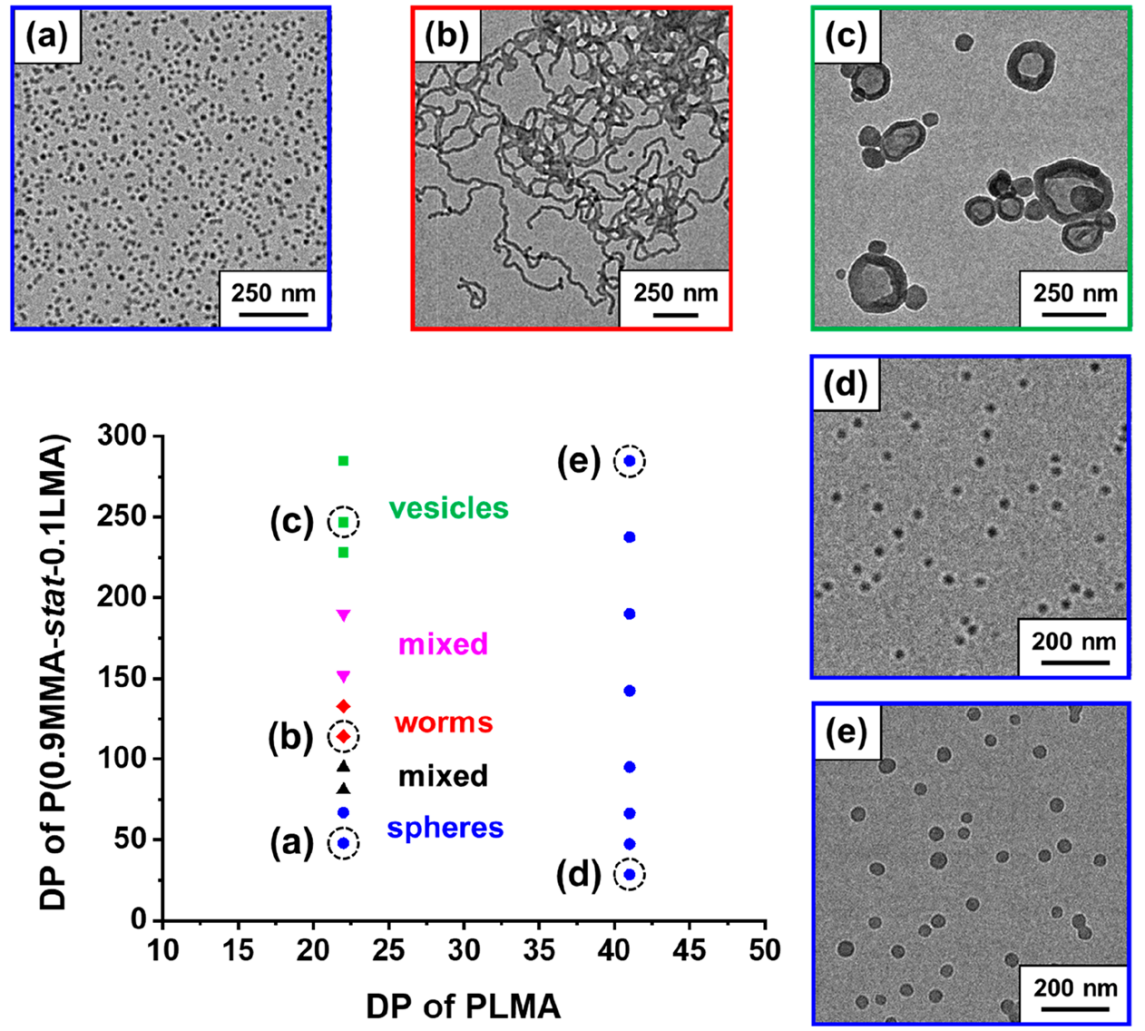
Pseudo-phase diagram constructed for PLMA_*x*_–P(0.9MMA-*stat*-0.1LMA)_*y*_ diblock copolymer nano-objects prepared by RAFT dispersion copolymerization of MMA with LMA ([MMA]/[LMA] molar ratio = 9.0) in mineral oil using either a PLMA_22_ or a PLMA_41_ precursor with DCP initiator at 115 °C ([PLMA_*x*_]/[DCP] molar ratio = 3.0). Representative TEM images obtained for (a) PLMA_22_–P(0.9MMA-*stat*-0.1LMA)_47_, (b) PLMA_22_–P(0.9MMA-*stat*-0.1LMA)_113_, (c) PLMA_22_–P(0.9MMA-*stat*-0.1LMA)_247_, (d) PLMA_41_–P(0.9MMA-*stat*-0.1LMA)_28_, and (e) PLMA_41_–P(0.9MMA-*stat*-0.1LMA)_282_ diblock copolymers at 20% w/w solids in mineral oil.

For PISA formulations, it is well-established that using a relatively short stabilizer block aids the formation of higher order morphologies (worms or vesicles).^17,22^ As mentioned above, a pseudo-phase diagram constructed for a series of PLMA_22_–PMMA_*y*_ nano-objects prepared at 20% solids in mineral oil showed that vesicles could not be accessed.^15^ Herein, the same PLMA_22_ precursor was used to target P(0.9MMA-*stat*-0.1LMA) DPs of 50–300 at 20% w/w solids in mineral oil at 115 °C (see Figure 5 and Table S1). Well-defined spheres with *z*-average diameters of 24 (PDI = 0.01) and 29 nm (PDI = 0.02) were obtained for P(0.9MMA-*stat*-0.1LMA) DPs of 47 or 66 (see Figure 5a). Transparent, free-standing gels were produced for DPs of 113 and 133, suggesting the presence of worms (see Figure 5b**)**. At DPs of 228 or above, vesicles were obtained in the form of highly turbid, free-flowing fluids, with *z*-average diameters ranging from 140 nm (PDI = 0.12) to 148 nm (PDI = 0.08) (see Figure 5c).

**Figure 6:**
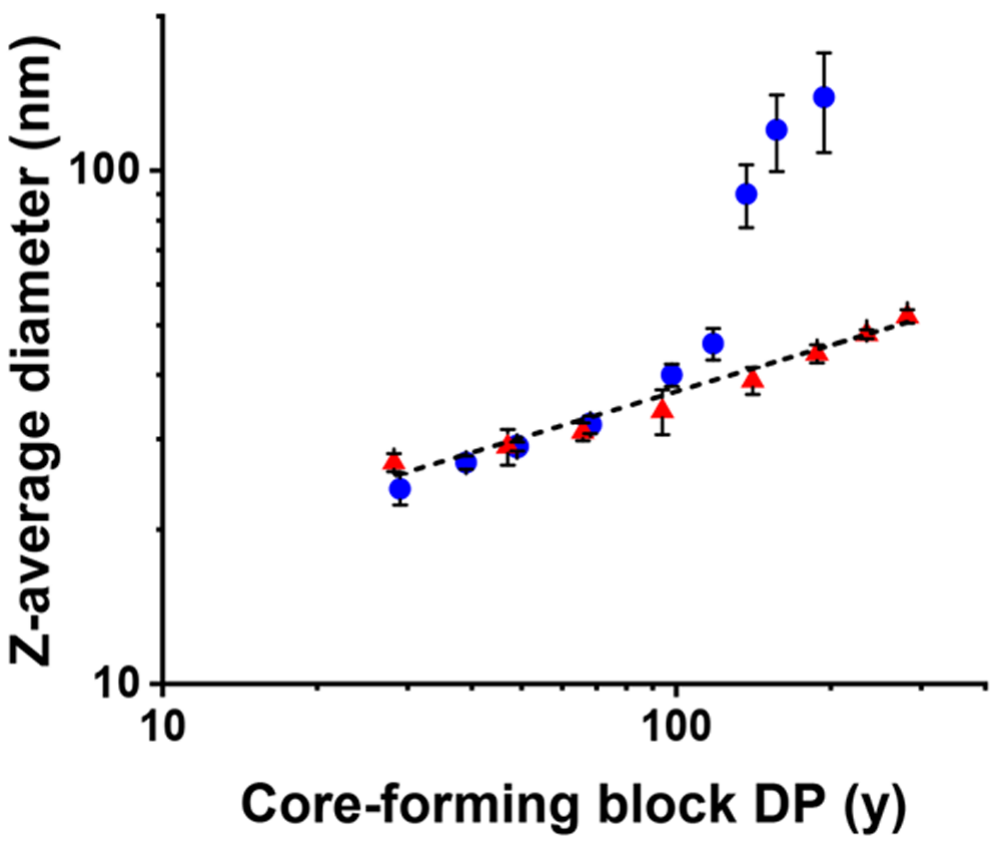
Double-logarithmic plot for the relationship between *z*-average diameter and core-forming block DP (*y*) for a series of PLMA_41_–PMMA_*y*_ (targeting *y* = 30–200) spheres prepared by RAFT dispersion polymerization of MMA at 90 °C (blue data) and a series of PLMA_41_–P(0.9MMA-*stat*-0.1LMA)_*y*_ (targeting *y* = 30–300) spheres prepared by RAFT dispersion copolymerization of LMA with MMA at 115 °C (red data) at 20% w/w solids in mineral oil. [N.B. Standard deviations are calculated from DLS polydispersities and thus indicate the breadth of the particle size distributions rather than the experimental error.] Blue data are taken from ref (15).

In contrast, only kinetically trapped spheres of increasing size were obtained when using a PLMA_41_ precursor to target a series of PLMA_41_–P(0.9MMA-*stat*-0.1LMA)_*y*_ nano-objects (*y* = 30–300) (see Figure 5d,e, Figure 6 (red data set), and Table S2). We have reported similar observations for various PISA syntheses conducted in non-polar media.^24,55^ For the analogous PLMA_41_–PMMA_*y*_ formulation, a linear relationship was initially observed between the DLS *z*-average diameter and PMMA DP.^15^ However, colloidally unstable aggregates were invariably obtained for PMMA DPs ≥ 137, as indicated by the substantial increase in particle size and PDI (see Figure 6 (blue data set)).^15^

**Figure 7:**
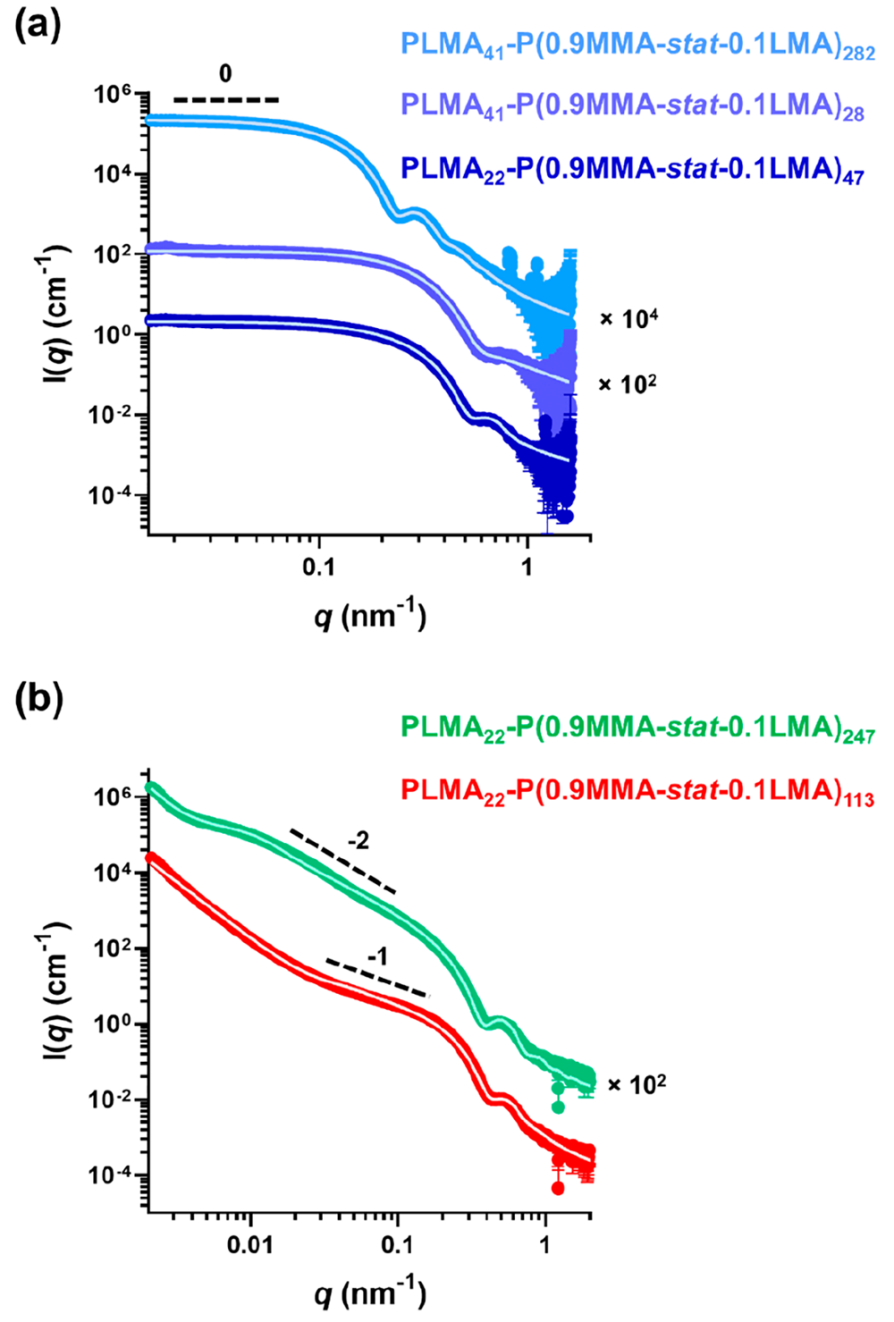
Small-angle X-ray scattering (SAXS) patterns and corresponding data fits (solid white lines) recorded for 1.0% w/w dispersions of (a) PLMA_22_–P(0.9MMA-*stat*-0.1LMA)_47_, PLMA_41_–P(0.9MMA-*stat*-0.1LMA)_28_, and PLMA_41_–P(0.9MMA-*stat*-0.1LMA)_282_ spheres and (b) PLMA_22_–P(0.9MMA-*stat*-0.1LMA)_113_ worms and PLMA_22_–P(0.9MMA-*stat*-0.1LMA)_247_ vesicles in mineral oil at 20 °C. These nano-objects were originally prepared at 115 °C targeting 20% w/w solids in mineral oil. Dashed lines are provided for guidance to the eye and indicate low *q* gradients of 0, −1, and −2.

To confirm the copolymer morphology assigned on the basis of TEM analysis (see Figure 5), small-angle X-ray scattering (SAXS) patterns were recorded for 1.0% w/w dispersions of five examples of PLMA_*x*_–P(0.9MMA-*stat*-LMA)_*y*_ nano-objects (see Figure 7). SAXS is much more statistically robust than TEM because such patterns are averaged over many millions of nanoparticles. It is well-known that the low *q* gradient in a SAXS pattern is diagnostic of the predominant copolymer morphology.^56^ Thus, spheres have a zero gradient, while worms and vesicles exhibit gradients of −1 and −2, respectively. Inspecting Figure 7, a low *q* gradient of zero was observed for PLMA_22_–P(0.9MMA-*stat*-0.1LMA)_47_, PLMA_41_–P(0.9MMA-*stat*-0.1LMA)_28_, and PLMA_41_–P(0.9MMA-*stat*-0.1LMA)_282_, while low *q* gradients of −1 and −2 were obtained for PLMA_22_–P(0.9MMA-*stat*-0.1LMA)_113_ and PLMA_22_–P(0.9MMA-*stat*-0.1LMA)_247_, respectively. Fitting the first three patterns (see Figure 7a) using a spherical micelle model^57^ indicated an overall volume-average diameter (*D*_sphere_) of 18.9 ± 1.9 nm and a mean aggregation number (*N*_agg_) of 200 for PLMA_22_–P(0.9MMA-*stat*-0.1LMA)_47_, a *D*_sphere_ of 19.8 ± 1.1 nm and an *N*_agg_ of 140 for PLMA_41_–P(0.9MMA-*stat*-0.1LMA)_28_, and a *D*_sphere_ of 43.0 ± 4.5 nm and an *N*_agg_ of 510 for PLMA_41_–P(0.9MMA-*stat*-0.1LMA)_282_ (see Table S3). These data are consistent with the corresponding *z*-average diameters reported by DLS, which were 24 nm (PDI = 0.01) for PLMA_22_–P(0.9MMA-*stat*-0.1LMA)_47_, 27 nm (PDI = 0.04) for PLMA_41_–P(0.9MMA-*stat*-0.1LMA)_28_ and 52 nm (PDI = 0.03) for PLMA_41_–P(0.9MMA-*stat*-0.1LMA)_282_ provided by DLS (see Tables S1 and S2).

SAXS patterns recorded for PLMA_22_–P(0.9MMA-*stat*-0.1LMA)_113_ and PLMA_22_–P(0.9MMA-*stat*-0.1LMA)_247_ are shown in Figure 7b. A satisfactory fit to the former pattern was obtained using a worm-like micelle model,^57^ which indicated a mean worm cross-sectional diameter of 20.0 ± 2.4 nm. The upturn in the scattering intensity observed in the low *q* region most likely indicates worm branching and/or clustering: unfortunately, this feature prevents determination of the mean contour length and a reliable *N*_agg_ for these worms. The pattern obtained for PLMA_22_–P(0.9MMA-*stat*-0.1LMA)_247_ could be satisfactorily fitted using a vesicle model,^58^ which indicated an overall volume-average diameter of 172 ± 126 nm, a mean vesicle membrane thickness of 15.4 ± 1.6 nm, and an *N*_agg_ of 25600. Comparing this aggregation number to that determined for the PLMA_22_–P(0.9MMA-*sta*t-0.1LMA)_47_ spheres, we calculate that the mean number of spheres that must undergo fusion to form a single vesicle is 128.^44^

### Thermoresponsive Behavior of PLMA_22_–P(0.9MMA-*stat*-0.1LMA)_113_ Worms and PLMA_22_–P(0.9MMA-*stat*-0.1LMA)_228_ Vesicles

**Figure 8:**
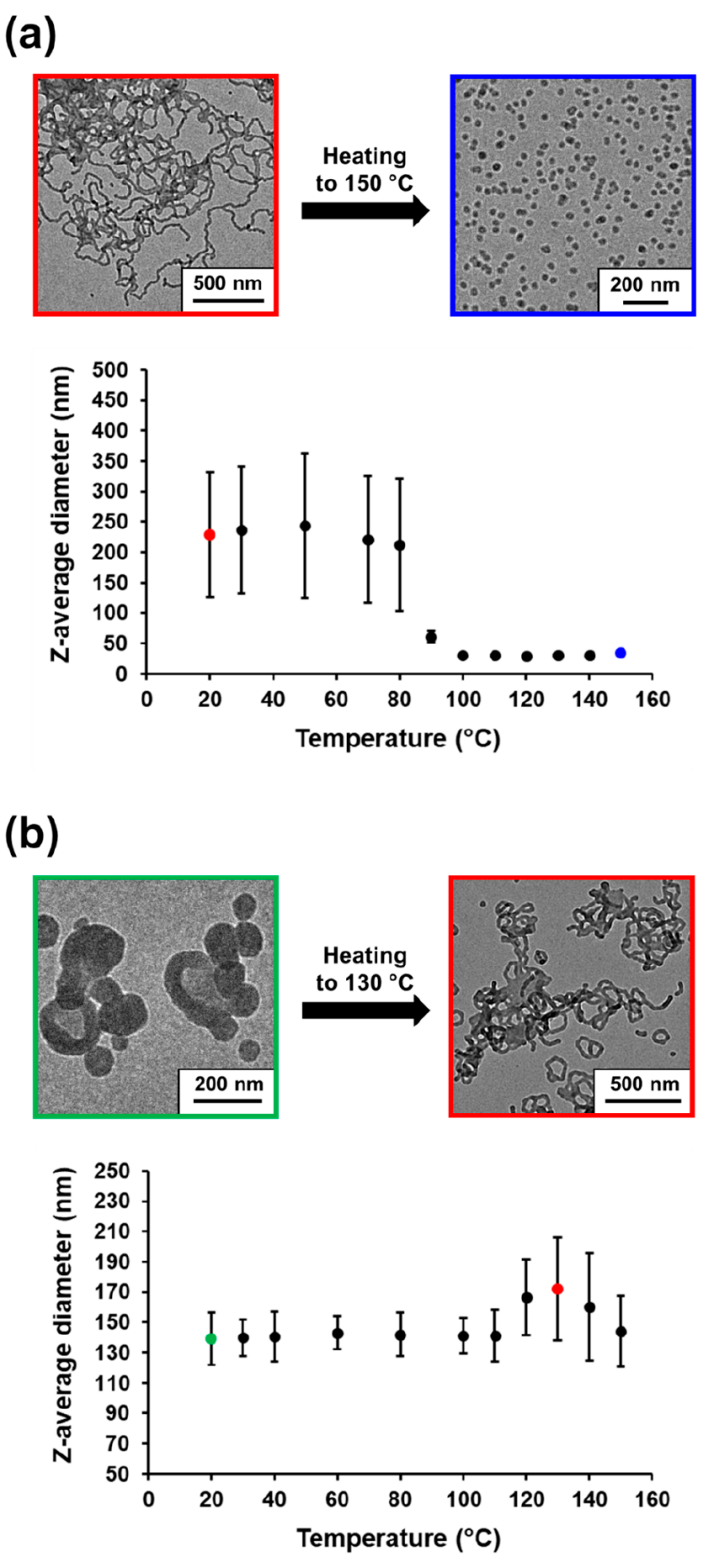
Variation in DLS *z*-average diameter observed for a 0.1% w/w dispersion of PLMA_22_–P(0.9MMA-*stat*-0.1LMA)_113_ nano-objects (prepared using *n*-dodecane as a diluent) on heating from 20 to 150 °C. Representative TEM images obtained for the initial worms (red frame) and the final spheres that are formed after heating to 150 °C (blue frame). (b) Variation in DLS *z*-average diameter for a 0.1% w/w PLMA_22_–P(0.9MMA-*stat*-0.1LMA)_228_ nano-objects (prepared using *n*-dodecane as a diluent) on heating from 20 to 150 °C. Representative TEM images obtained for the initial vesicles (green frame) and the worms formed after heating to 130 °C (red frame). [N.B. Standard deviations are calculated from the DLS polydispersity data and indicate the breadth of the particle size distribution, rather than the experimental error.]

**Figure 9:**
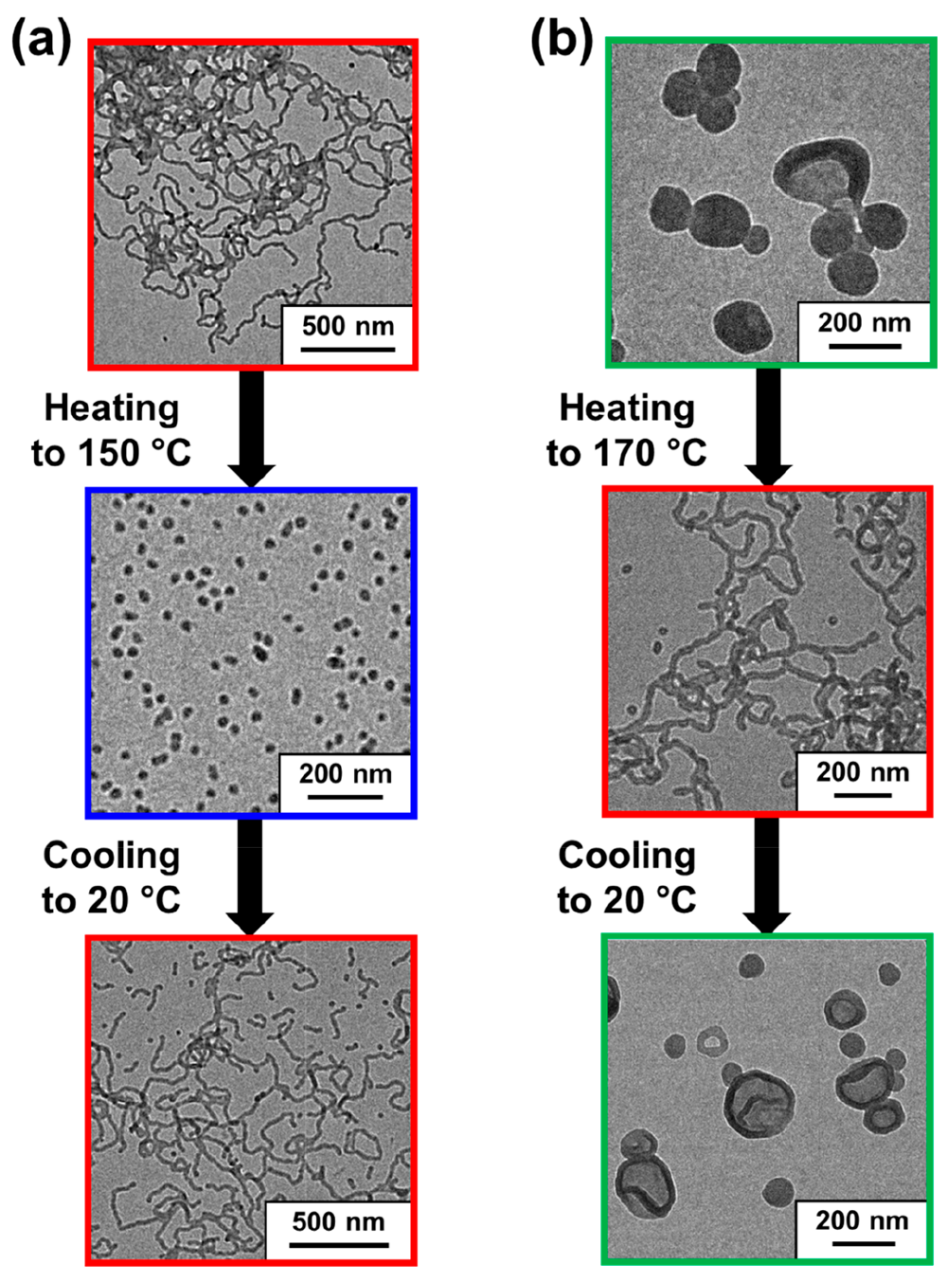
(a) Representative TEM images recorded during the worm-to-sphere transition for PLMA_22_–P(0.9MMA-*stat*-0.1LMA)_113_ worms prepared at 20% w/w solids in mineral oil. The initial copolymer dispersion (red frame) was heated to 150 °C and equilibrated for 30 min at this temperature, prior to dilution with hot *n*-dodecane (blue frame) and finally aged for 24 h at 20 °C (red frame). (b) Representative TEM images recorded during the vesicle-to-worm transition for PLMA_22_–P(0.9MMA-*stat*-0.1LMA)_228_ vesicles prepared at 20% w/w solids in mineral oil. The initial copolymer dispersion (green frame) was heated to 170 °C and equilibrated for 30 min at this temperature, prior to dilution with hot *n*-dodecane (red frame) and finally aged for 24 h at 20 °C (green frame).

The PISA literature contains examples of thermoresponsive diblock copolymer nano-objects prepared in non-polar media, with both worm-to-sphere transitions^15,36,59,60^ and vesicle-to-worm transitions^44,61^ being observed at elevated temperature. Such transitions can be explained in terms of surface plasticization of the insoluble block by ingress of hot solvent.^36,44^ The worm-to-sphere transition is typically reversible for a concentrated copolymer dispersion (e.g., 5–20% solids) but becomes irreversible at the relatively high dilution (e.g., 0.1% w/w) typically used for DLS experiments.^36^ This is because of the reduced probability of efficient interparticle fusion to reform worms, which is a highly cooperative process.^36^ In our recent PLMA–PMMA study,^15^ we used DLS to examine the thermoresponsive behavior of PLMA_22_–PMMA_69_ short worms, which exhibited an irreversible worm-to-sphere transition on heating above 110 °C. For this system, only partial reversibility was observed by TEM at 20% w/w solids after a 20 °C to 150 °C to 20 °C thermal cycle.^15^ Herein, we explore the thermoresponsive behavior of PLMA_22_–P(0.9MMA-*stat*-0.1LMA)_113_ worms and PLMA_22_–P(0.9MMA-*stat*-0.1LMA)_228_ vesicles (see Table S1) via DLS and TEM studies.^15^ These compositions were selected because they lie close to the phase boundaries indicated in Figure 5, which should enhance the probability of observing the expected thermal transition.^44^ For DLS experiments, the as-synthesized 20% w/w dispersions of PLMA_22_–P(0.9MMA-*stat*-0.1LMA)_113_ worms and PLMA_22_–P(0.9MMA-*stat*-0.1LMA)_228_ vesicles in mineral oil were diluted to 0.1% w/w using *n*-dodecane. The latter solvent was chosen as a diluent to facilitate TEM grid preparation because it is much more volatile than mineral oil. The resulting dilute copolymer dispersions were heated from 20 to 150 °C, with equilibration for 30 min at 10 °C intervals prior to extracting each aliquot. Aliquots were then cooled to 20 °C prior to DLS studies and subsequent TEM analysis. This “cumulative thermal annealing” protocol was adopted because the upper limit temperature for our DLS instrument is only 90 °C, which is insufficient to produce the desired thermal transition (see Figure 8). For this experimental protocol, an implicit assumption is that the copolymer morphology accessed at high temperature is retained on cooling to 20 °C because high dilution ensures that the thermal transition is essentially irreversible. The sphere-equivalent *z*-average diameter observed for PLMA_22_–P(0.9MMA-*stat*-0.1LMA)_113_ worms remained almost unchanged between 20 and 80 °C with minimal variation in the associated DLS polydispersity (PDI) (see Figure 8a). A substantial reduction in both parameters occurs on further heating from 80 °C (213 nm; PDI = 0.51) to 100 °C (31 nm; PDI = 0.05). These data suggest a worm-to-sphere transition within this temperature range, which was confirmed by TEM analysis (see Figure 8a). For the PLMA_22_–P(0.9MMA-*stat*-0.1LMA)_228_ vesicles, an apparent increase in the *z*-average diameter and PDI was observed on raising the temperature from 110 °C (141 nm; PDI = 0.12) to 130 °C (172 nm; PDI = 0.20) (see Figure 8b). TEM analysis indicated the formation of branched worms (see Figure 8b).^44^ Further heating to 140 °C led to a modest reduction in the *z*-average diameter (160 nm; PDI = 0.22) and the formation of mainly worms plus a minor population of spheres (see Figure S5). TEM was used to study the reversibility of these thermal transitions for 20% w/w copolymer dispersions. Accordingly, PLMA_22_–P(0.9MMA-*stat*-0.1LMA)_113_ worms and PLMA_22_–P(0.9MMA-*stat*-0.1LMA)_228_ vesicles were each heated in turn to 150 °C and equilibrated for 1 h. Then a small aliquot was extracted in each case and immediately diluted to 0.1% w/w by using hot *n*-dodecane (preheated to 150 °C) to assess the copolymer morphology. The PLMA_22_–P(0.9MMA-*stat*-0.1LMA)_113_ worms were converted into spheres on heating (see Figure 9a). Only “jellyfish” structures were formed on heating the PLMA_22_–P(0.9MMA-*stat*-0.1LMA)_228_ vesicles to 150 °C (see Figure S6), but further heating to 170 °C generated a pure worm morphology (see Figure 9b). Thereafter, both 20% w/w copolymer dispersions were allowed to cool to 20 °C and stored at this temperature for 24 h prior to dilution with *n*-dodecane.^15^ Subsequent TEM analysis confirmed that both thermal transitions were more or less reversible for such concentrated dispersions (Figure 9) with the corresponding DLS data supporting these observations (Table S4).

## Conclusions

Recently, we reported the synthesis of PLMA_22_–PMMA_*y*_ diblock copolymer nanoparticles in mineral oil but only spheres, short worms, or micrometer-sized spherical aggregates could be produced. To overcome this unexpected morphological constraint, a series of PLMA_*x*_–P(0.9MMA-*stat*-0.1LMA)_*y*_ diblock copolymer nanoparticles were prepared via RAFT dispersion copolymerization of MMA with 10 mol % LMA at 20% solids in mineral oil using either PLMA_22_ or PLMA_41_ precursors at 115 °C. *In situ*^1^H NMR studies indicated an overall comonomer conversion of 94% within 2.5 h when targeting a core-forming block DP of 300. A narrow molecular weight distribution (*M*_w_/*M*_n_ ≤ 1.35) was confirmed for each diblock copolymer. Systematic variation of the LMA content from 0 to 10 mol % resulted in a gradual reduction in the *T*_g_ of the core-forming block. Empirically, it was found that at least 10 mol % LMA was required to promote the formation of higher order morphologies for syntheses performed at 115 °C. Using the relatively short PLMA_22_ precursor enabled pure spheres, worms, or vesicles to be obtained, as judged by TEM, DLS, and SAXS analysis. Furthermore, a worm-to-sphere transition was observed for PLMA_22_–P(0.9MMA-*stat*-0.1LMA)_113_ worms at elevated temperature while PLMA_22_–P(0.9MMA-*stat*-0.1LMA)_228_ vesicles underwent a vesicle-to-worm transition on heating. DLS studies indicated that such morphology transitions were irreversible for 0.1% w/w dispersions, but good reversibility was observed for 20% w/w dispersions according to TEM and DLS analysis.

## Supporting Information Available

Experimental section; assigned ^1^H NMR spectra for PLMA_22_–P(0.9MMA-*stat*-0.1LMA)_282_ diblock copolymer; DSC thermogram of PLMA_22_; TEM image of the PLMA_22_–P(0.9MMA-*stat*-0.1LMA)_282_ vesicles prepared during the kinetic experiment; TEM, GPC, and NMR data suggesting that LMA does not plasticize PMMA; summary tables of copolymer characterization data, summary table of SAXS fitting parameters; TEM image of worms obtained when heating 0.1% w/w PLMA_22_–P(0.9MMA-*stat*-0.1LMA)_228_ vesicle dispersion to 140 °C; TEM evidence for the partial vesicle-to-worm transition of 20% w/w PLMA_22_–P(0.9MMA-*stat*-0.1LMA)_228_ vesicles when heating to 150 °C; summary of tabulated DLS data obtained for PLMA_22_–P(0.9MMA-*stat*-0.1LMA)_113_ nanoparticles during a 20 °C to 150 °C to 20 °C thermal cycle and PLMA_22_–P(0.9MMA-*stat*-0.1LMA)_228_ nanoparticles during a 20 °C to 170 °C to 20 °C thermal cycle (PDF)
