# Supplementary material for: Tuning the Glass Transition Temperature of a Core-Forming Block during Polymerization-Induced Self-Assembly: Statistical Copolymerization of Lauryl Methacrylate with Methyl Methacrylate Provides Access to Spheres, Worms, and Vesicles
Source: Macromolecules. 2022 May 11;55(10):4091–101. doi: 10.1021/acs.macromol.2c00475 (PMC9134497; doi:10.1021/acs.macromol.2c00475)
Supplement: Supplementary file 1 — ma2c00475_si_001.pdf [file ma2c00475_si_001.pdf]

Supporting Information for:

Tuning the Glass Transition Temperature of a Core-Forming Block During Polymerization-Induced Self-Assembly: Statistical Copolymerization of Lauryl Methacrylate with Methyl Methacrylate Provides Access to Spheres, Worms and Vesicles

Csilla György, Thomas J. Neal, Timothy Smith, David J. Growney and Steven P. Armes*

Table of Contents

Experimental Section S2

Figure S1. ^{1}H NMR spectra for targeting $PLMA_{22}$-P(0.9MMA-*stat*-$0.1LMA)_{300}$ composition at 20% w/w solids in mineral oil S7

Figure S2. DSC curve of $PLMA_{22}$ precursor S8

Figure S3. Representative TEM image obtained for the $PLMA_{22}$-P(0.9MMA-*stat*-$0.1LMA)_{282}$ vesicles prepared during the *in situ* ^{1}H NMR kinetic experiment S8

Figure S4. TEM, GPC and NMR data obtained for the $PLMA_{22}$-$PMMA_{291}$ block copolymer after heating with excess LMA at 115 °C for 17 h S9

Table S1. Summary of data obtained for a series of $PLMA_{22}$-P(MMA-*stat*-$LMA)_y$ nano-objects prepared at 115 °C S10

Table S2. Summary of data obtained for a series of $PLMA_{41}$-P(0.9MMA-*stat*-$0.1LMA)_y$ nano-objects prepared at 115 °C S10

Table S3. Summary table of SAXS fitting parameters S11

Figure S5. Representative TEM image recorded for the 0.1% w/w $PLMA_{22}$-P(0.9MMA-*stat*-$0.1LMA)_{228}$ dispersion after heating to 140 °C for the DLS study S11

Figure S6. Representative TEM image recorded for $PLMA_{22}$-P(0.9MMA-*stat*-$0.1LMA)_{228}$ diblock copolymer after heating the 20% w/w dispersion to 150 °C S12

Table S4. Summary of DLS data obtained for the worm-to-sphere transition of $PLMA_{22}$-P(0.9MMA-co-$0.1LMA)_{113}$ worms and for the vesicle-to-worm transition of the $PLMA_{22}$-P(0.9MMA-co-$0.1LMA)_{228}$ vesicles S12

References S13

Experimental Section

Materials

Methyl methacrylate (MMA, 99%) was purchased from Alfa Aesar (Germany), passed through basic alumina to remove its inhibitor and then stored at –20 °C prior to use. Lauryl methacrylate (LMA), dicumyl peroxide (DCP), $CDCl_3$, ruthenium(IV) oxide, sodium periodate and *n*-dodecane were purchased from Merck (UK) and used as received. 2,2'-Azoisobutyronitrile (AIBN) was obtained from Molekula (UK) and *tert*-butyl peroxy-2-ethylhexanoate (T21s) was purchased from AkzoNobel (The Netherlands). CD_2Cl_2 was purchased from Goss Scientific (UK). Tetrahydrofuran was obtained from VWR Chemicals (UK). Methanol and toluene were purchased from Fisher Scientific (UK). 4-Cyano-4(dodecylthiocarbonothioylthio)pentanoate (MCDP) and Group III hydroisomerized mineral oil (viscosity = 4.3 cSt at 100 °C) were kindly provided by The Lubrizol Corporation Ltd. (Hazelwood, Derbyshire, UK).

Synthesis of PLMA precursors *via* RAFT solution polymerization in toluene

$PLMA_{22}$ and $PLMA_{41}$ precursors were synthesized at 50% w/w solids using the previously reported synthesis protocol.[1] A typical synthesis of $PLMA_{22}$ was conducted as follows. LMA (48.7 g; 191.5 mmol), MCDP (4.00 g; 9.6 mmol; target DP = 20), AIBN (315 mg; 1.9 mmol; MCDP/AIBN molar ratio = 5.0) and toluene (53.0 g) were weighed into a 250 mL round-bottomed flask. The sealed flask was purged with nitrogen for 30 min and immersed in a preheated oil bath at 80 °C. The reaction solution was stirred continuously and the ensuing polymerization was quenched after 4.5 h by exposing the reaction solution to air and cooling the flask to room temperature. A final LMA conversion of 91% was determined by ^1H NMR spectroscopy. The crude polymer was purified by three consecutive precipitations into a ten-fold excess of methanol (with redissolution in THF after each precipitation). The mean DP of this PLMA precursor was calculated to be 22 by using ^1H NMR spectroscopy to compare the

three methyl protons assigned to the methyl ester end-group at 3.7 ppm to the two oxymethylene protons attributed to PLMA at 3.80–4.20 ppm. THF GPC analysis using a refractive index detector and a series of near-monodisperse poly(methyl methacrylate) calibration standards indicated an M_n of 6 000 g mol^{-1} and an M_w/M_n of 1.13. The theoretical M_n of the $PLMA_{22}$ precursor is 6014.71 g mol^{-1}. Thus, despite the large difference in molar mass between LMA and MMA, a remarkably small calibration error is incurred when using PMMA standards to analyse PLMA homopolymers by THF GPC. This suggests that minimal calibration errors should be incurred when analysing the $PLMA_{22}$-P(0.9MMA-*stat*-$0.1LMA)_x$ diblock copolymers reported in the present study.

Synthesis of poly(lauryl methacrylate)-poly(methyl methacrylate-*stat*-lauryl methacrylate) ($PLMA_{22}$-P(0.9MMA-*stat*-$0.1LMA)_{282}$) diblock copolymer nanoparticles *via* RAFT dispersion copolymerization of LMA with MMA in mineral oil

The following example is representative for targeting $PLMA_{22}$-P(0.9MMA-*stat*-$0.1LMA)_{300}$ nanoparticles at 20% w/w solids. $PLMA_{22}$ precursor (0.20 g; 33.25 µmol), LMA monomer (0.25 g; 997.55 µmol), DCP initiator (3.0 mg; 11.08 µmol) and mineral oil (5.42 g) were weighed into a glass vial and purged with nitrogen for 30 min. MMA monomer (0.96 mL; 8.98 mmol) was degassed separately then added to the reaction mixture *via* syringe. The sealed vial was immersed in a preheated oil bath at 115 °C and the reaction mixture was magnetically stirred for 17 h. ^{1}H NMR analysis indicated an overall comonomer conversion of 94% by comparing the integrated vinyl signals observed for the final reaction mixture to the methoxy signals assigned to the MMA and PMMA at 3.50-3.79 ppm while allowing for the initial [MMA]/[LMA] molar ratio of 9:1 (see **Figure S1**). An MMA conversion of 96% was determined by comparing the integrated methyl signal assigned to MMA monomer at 3.75–3.79 ppm to that of the copolymer at 3.50–3.72 ppm at the end of the copolymerization. An

LMA conversion of 76% was determined by comparing the integrated methylene signal for LMA monomer at 4.12–4.19 ppm to the combined methoxy signal for both MMA and PMMA according to the initial [MMA]/[LMA] molar ratio. THF GPC analysis indicated an M_n of 34 800 g mol^{-1} and an M_w/M_n of 1.25. To construct a pseudo-phase diagram for PLMA$_x$-P(0.9MMA-*stat*-0.1LMA)$_y$ nano-objects, a range of diblock copolymer compositions were targeted using PLMA$_{22}$ and PLMA$_{41}$ precursors in mineral oil at 20% w/w solids (see **Table S1** and **S2**). In each case, the same mass of PLMA$_x$ precursor was employed and the [0.9MMA-*stat*-0.1LMA]/[PLMA$_x$] molar ratio and the volume of mineral oil were adjusted accordingly. Since these copolymerizations were performed at 115 °C, the round-bottomed flask was sealed with a plastic cap rather than a rubber septum in order to prevent evaporative loss of MMA monomer (boiling point = 101 °C). It is important to note that conducting polymerizations above the boiling point of the MMA monomer can be potentially hazardous: scale-up syntheses of these nanoparticles would most likely require a pressurized reactor. PLMA$_{22}$-PMMA$_{300}$ and PLMA$_{22}$-P(0.9MMA-*stat*-0.1LMA)$_{300}$ diblock copolymers were also targeted at 90 °C using T21s initiator (dissolved at 10% v/v in mineral oil; [T21s]/[PLMA$_{22}$] = 3.0) at 20% w/w solids in mineral oil.

^{1}H NMR Spectroscopy

^{1}H NMR spectra were recorded in either CD_2Cl_2 or $CDCl_3$ using a 400 MHz Bruker Avance spectrometer. Typically, 64 scans were averaged per spectrum. *In situ* ^{1}H NMR spectra were recorded using the same spectrometer to study the kinetics of the synthesis of P(0.9MMA-*stat*-0.1LMA)$_{282}$ vesicles at 20% w/w solids in mineral oil. A 0.20 mL aliquot of the reaction mixture was transferred into an NMR tube equipped with a J-Young’s tap under an inert nitrogen atmosphere. A capillary tube containing 0.17 M benzylamine dissolved in d$_6$-DMSO was flame-sealed and used as an external standard (and also a solvent lock). A reference spectrum was recorded at 20 °C prior to heating the reaction mixture to 115 °C. Spectra were

acquired in 16 transients using a 30° excitation pulse and a delay time of 1 s over a spectral window of 8 kHz with 64 k data points and recorded approximately every 10 min for 2.5 h.

Gel Permeation Chromatography (GPC)

Molecular weight distributions (MWDs) were assessed by GPC using THF as an eluent. The GPC system was equipped with two 5 μm (30 cm) Mixed C columns and a WellChrom K-2301 refractive index detector operating at 950 ± 30 nm. The THF mobile phase contained 2.0% v/v triethylamine and 0.05% w/v butylhydroxytoluene (BHT) and the flow rate was fixed at 1.0 ml min^{-1}. A series of twelve near-monodisperse poly(methyl methacrylate) standards (M_p values ranging from 800 to 2 200 000 g mol^{-1}) were used for column calibration in combination with a refractive index detector.

Dynamic Light Scattering (DLS)

DLS studies were performed using a Zetasizer Nano ZS instrument (Malvern Instruments, UK) at a fixed scattering angle of 173°. Copolymer dispersions were diluted in *n*-dodecane (0.10% w/w) prior to light scattering studies at 20 °C. The intensity-average diameter and polydispersity of the nanoparticles were calculated by cumulants analysis of the experimental correlation function using Dispersion Technology Software version 6.20. Data were averaged over ten runs each of thirty seconds duration. It is emphasized that DLS assumes a spherical morphology. Thus, the DLS diameter calculated for anisotropic nanoparticles such as worms is a 'sphere-equivalent' value that indicates neither the worm length nor the worm width. Nevertheless, DLS can be used to monitor a thermally-induced worm-to-sphere transition by determining the reduction in the apparent diameter as a function of temperature.[2]

Transmission Electron Microscopy (TEM)

TEM studies were conducted using a Philips CM 100 instrument operating at 100 kV and equipped with a Gatan 1k CCD camera. A single droplet of a 0.10% w/w copolymer dispersion was placed onto a carbon-coated copper grid and allowed to dry, prior to exposure to ruthenium(VIII) oxide vapor for 7 min at 20 °C.[3] This heavy metal compound acts as a positive stain for the core-forming PMMA block to improve contrast. The ruthenium(VIII) oxide was prepared as follows: ruthenium(IV) oxide (0.30 g) was added to water (50 g) to form a black slurry; addition of sodium periodate (2.0 g) with continuous stirring produced a yellow solution of ruthenium(VIII) oxide within 1 min at 20 °C.

Small-angle X-ray scattering (SAXS)

SAXS patterns were collected at a synchrotron source (ESRF, beamline ID02, Grenoble, France; experiment number SC-5109) using a monochromatic X-ray radiation (wavelength λ = 0.0995 nm, with q ranging from 0.0021 to 2.0 nm^{-1}, where $q = (4\pi/\lambda)$. sinθ is the length of the scattering vector and θ is the one-half of the scattering angle) and a Ravonix MX-170HS CCD detector. A glass capillary of 2 mm diameter was used as a sample holder. Scattering data were reduced using standard routines from the beamline[4] and were further analyzed using Irena SAS macros for Igor Pro.[5]

Differential scanning calorimetry (DSC)

Measurements were performed using a TA DSC25 Discovery series instrument operating from -90 to 180 °C at a rate of 5 °C min^{-1} using aluminum T_{zero} pans and T_{zero} hermetic lids for $PLMA_{22}$, $PLMA_{22}$-$PMMA_{192}$, $PLMA_{22}$-P(0.95MMA-*stat*-0.05LMA)$_{188}$, $PLMA_{22}$-P(0.9MMA-*stat*-0.1LMA)$_{188}$ and $PLMA_{22}$-P(0.8MMA-*stat*-0.2LMA)$_{186}$. Instrument calibration was performed using an indium standard. Purified copolymer powders were obtained after three consecutive precipitations of the as-synthesized diblock copolymer

dispersion into a ten-fold excess of methanol (with redissolution in THF after each precipitation), followed by isolation *via* filtration and drying under vacuum for 24 h. For DSC analysis, the $PLMA_{22}$ precursor and each diblock copolymer were subjected to two heating/cooling cycles: the first cycle ensured removal of residual organic solvent, and the glass transition temperature was determined during the second cycle.

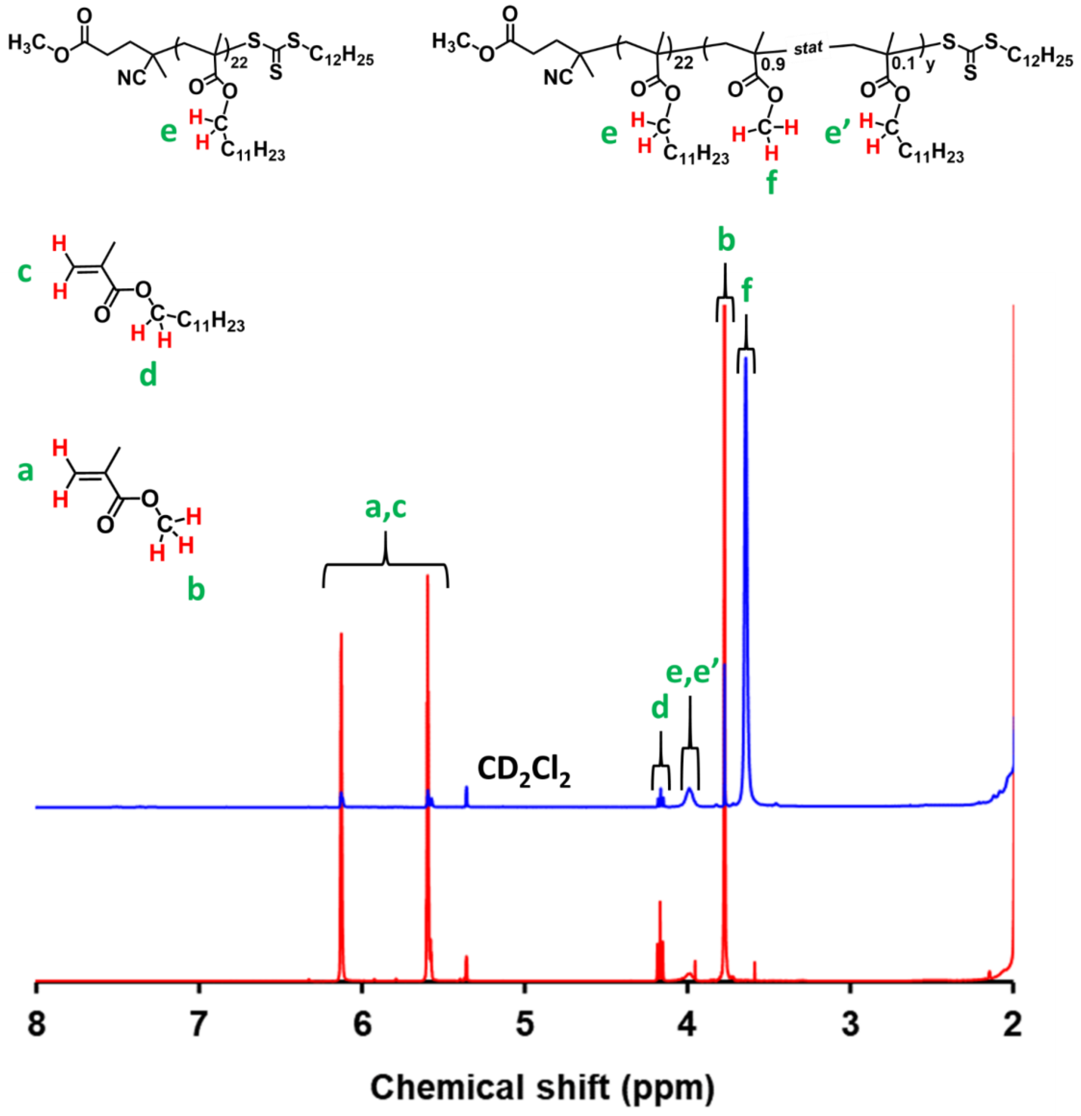

Figure S1. Assigned ^{1}H NMR spectra recorded in CD_2Cl_2 when targeting $PLMA_{22}$-$P(0.9MMA$-*stat*-$0.1LMA)_{300}$ at 20% w/w solids in mineral oil. Initial reaction mixture (red spectrum) and final reaction mixture after 17 h at 115 °C (blue spectrum). The overall comonomer conversion was determined to be 94% while the final MMA and LMA conversions were 96% and 76%, respectively.

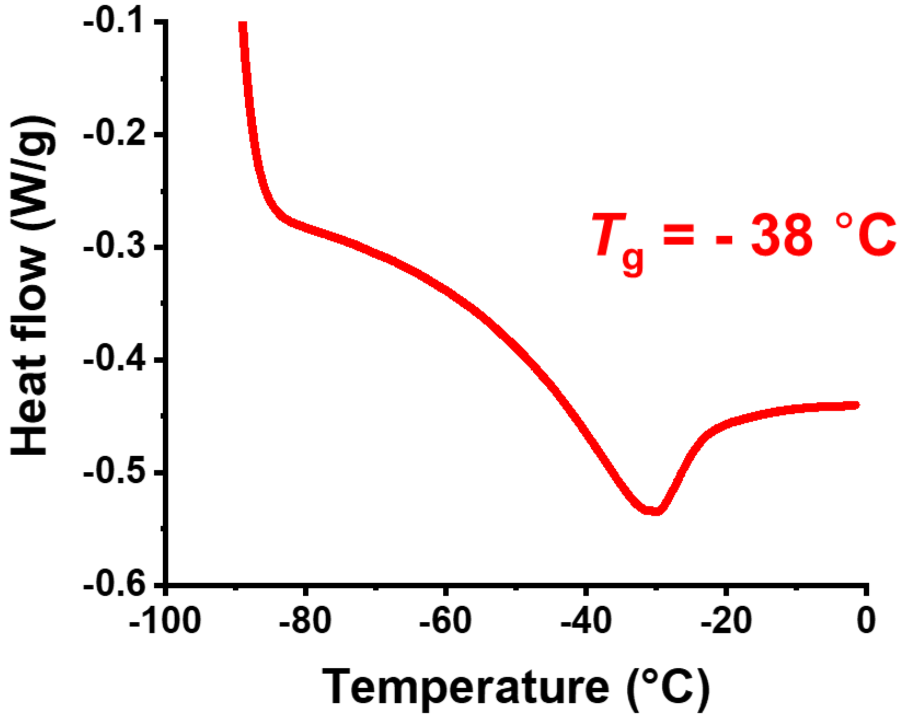

Figure S2. DSC curve recorded for the PLMA$_{22}$ precursor, for which the T_g was determined to be –38 °C.

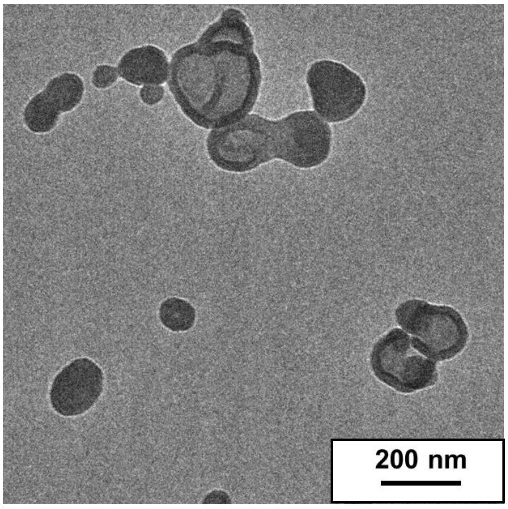

Figure S3. Representative TEM image obtained for the final PLMA$_{22}$-P(0.9MMA-*stat*-0.1LMA)$_{282}$ vesicles after 3 h when targeting 20% w/w solids in mineral oil at 115 °C during the *in situ* ^{1}H NMR kinetic experiment.

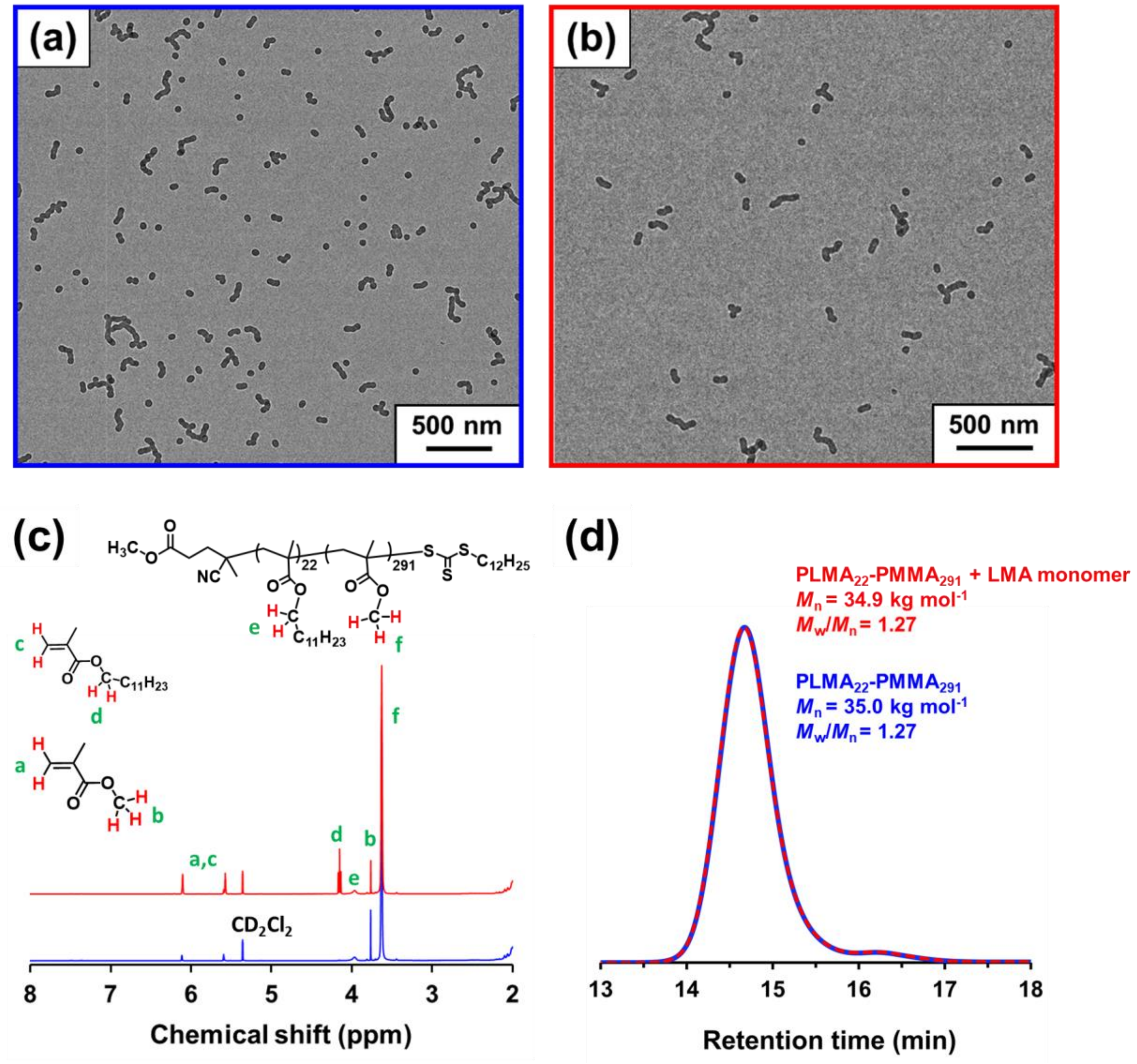

Figure S4. Representative TEM images obtained for **(a)** $PLMA_{22}$-$PMMA_{291}$ nano-objects prepared at 20% w/w solids in mineral oil at 115 °C and **(b)** the same diblock copolymer after heating the 20% w/w dispersion at 115 °C for 17 h in the presence of a large excess of LMA monomer ([LMA]/[$PLMA_{22}$-$PMMA_{291}$] molar ratio = 50). **(c)** Assigned ^{1}H NMR spectra recorded in CD_2Cl_2 for the initial $PLMA_{22}$-$PMMA_{291}$ diblock copolymer (blue spectrum) and that obtained after thermal annealing at 115 °C for 17 h (red spectrum). **(d)** GPC traces recorded using a refractive index detector (and expressed relative to a series of near-monodisperse poly(methyl methacrylate) calibration standards) for the initial $PLMA_{22}$-$PMMA_{291}$ diblock copolymer (blue curve) and the copolymer obtained after thermal annealing at 115 °C for 17 h (red data). Clearly, no LMA polymerization occurs during thermal annealing.

Table S1. Summary of the overall comonomer conversion (determined by ^{1}H NMR analysis), GPC, DLS and TEM data obtained for a series of $PLMA_{22}$-P(0.9MMA-*stat*-0.1LMA)$_y$ nano-objects prepared at 20% w/w solids in mineral oil at 115 °C. The $PLMA_{22}$ precursor block is also included as a reference.

Target Composition	Comonomer Conversion (%)	THF GPC		DLS		TEM Morphology
		M_n (g mol^{-1})	M_w/M_n	D_h (nm)	PDI	
$PLMA_{22}$ precursor	-	6 000	1.13	-	-	-
$PLMA_{22}$-P(0.9MMA-*stat*-0.1LMA)$_{50}$	94	13 800	1.16	24	0.01	Spheres
$PLMA_{22}$-P(0.9MMA-*stat*-0.1LMA)$_{70}$	94	14 800	1.17	29	0.02	Spheres
$PLMA_{22}$-P(0.9MMA-*stat*-0.1LMA)$_{85}$	94	16 700	1.18	54	0.14	Spheres and Worms
$PLMA_{22}$-P(0.9MMA-*stat*-0.1LMA)$_{100}$	94	17 200	1.19	82	0.15	Spheres and Worms
$PLMA_{22}$-P(0.9MMA-*stat*-0.1LMA)$_{120}$	94	19 200	1.21	229	0.45	Worms
$PLMA_{22}$-P(0.9MMA-*stat*-0.1LMA)$_{140}$	95	21 800	1.20	269	0.44	Worms
$PLMA_{22}$-P(0.9MMA-*stat*-0.1LMA)$_{160}$	95	25 200	1.20	2169	0.80	Worms and Vesicles
$PLMA_{22}$-P(0.9MMA-*stat*-0.1LMA)$_{200}$	94	26 400	1.25	142	0.14	Worms and Vesicles
$PLMA_{22}$-P(0.9MMA-*stat*-0.1LMA)$_{240}$	95	31 000	1.22	140	0.12	Vesicles
$PLMA_{22}$-P(0.9MMA-*stat*-0.1LMA)$_{260}$	95	34 000	1.25	141	0.11	Vesicles
$PLMA_{22}$-P(0.9MMA-*stat*-0.1LMA)$_{300}$	94	34 800	1.25	148	0.08	Vesicles
$PLMA_{22}$-P(0.95MMA-*stat*-0.05LMA)$_{200}$	94	25 000	1.20	54	0.04	Spheres
$PLMA_{22}$-P(0.8MMA-*stat*-0.2LMA)$_{200}$	93	29 400	1.22	2140	0.32	Large compound vesicles

Table S2. Summary of the overall comonomer conversion (determined by ^{1}H NMR analysis), GPC, DLS and TEM data obtained for a series of $PLMA_{41}$-P(0.9MMA-*stat*-0.1LMA)$_y$ nano-objects prepared at 20% w/w solids in mineral oil at 115 °C. The $PLMA_{41}$ precursor block is also included as a reference.

Target Composition	Comonomer Conversion (%)	THF GPC		DLS		TEM Morphology
		M_n (g mol^{-1})	M_w/M_n	D_h (nm)	PDI	
$PLMA_{41}$ precursor	-	11 300	1.12	-	-	-
$PLMA_{41}$-P(0.9MMA-*stat*-0.1LMA)$_{30}$	94	17 400	1.17	27	0.04	Spheres
$PLMA_{41}$-P(0.9MMA-*stat*-0.1LMA)$_{50}$	94	18 900	1.19	29	0.08	Spheres
$PLMA_{41}$-P(0.9MMA-*stat*-0.1LMA)$_{70}$	94	20 000	1.21	31	0.04	Spheres
$PLMA_{41}$-P(0.9MMA-*stat*-0.1LMA)$_{100}$	94	21 700	1.21	34	0.10	Spheres
$PLMA_{41}$-P(0.9MMA-*stat*-0.1LMA)$_{150}$	94	27 500	1.30	39	0.06	Spheres
$PLMA_{41}$-P(0.9MMA-*stat*-0.1LMA)$_{200}$	94	32 900	1.35	44	0.04	Spheres
$PLMA_{41}$-P(0.9MMA-*stat*-0.1LMA)$_{250}$	94	37 400	1.33	48	0.02	Spheres
$PLMA_{41}$-P(0.9MMA-*stat*-0.1LMA)$_{300}$	94	40 000	1.35	52	0.03	Spheres

Table S3. Summary of the structural parameters obtained from fitting SAXS patterns recorded for a series of $PLMA_x$-P(0.9MMA-*stat*-0.1LMA)$_y$ nano-objects using a spherical micelle[6], a worm-like micelle model[6] or a vesicle model[7]. D_{sphere} is the overall sphere diameter such that $D_{sphere} = 2R_s + 4R_g$, where R_s is the mean core radius and R_g is the radius of gyration of the stabilizer chains. T_{worm} is the overall worm thickness ($T_{worm} = 2R_{wc} + 4R_g$, where R_{wc} is the mean worm core radius) and L_{worm} is the mean worm contour length. $D_{vesicle}$ is the overall vesicle diameter ($D_{vesicle} = 2R_m + T_{membrane} + 4R_g$, where R_m is the distance from the centre of the vesicle to the centre of the vesicle membrane, and $T_{membrane}$ is the vesicle membrane thickness). N_{agg} is the mean aggregation number (i.e. the mean number of copolymer chains per nano-object).

Block Copolymer	Nanoparticle Morphology	D_{sphere} (nm)	T_{worm} (nm)	L_{worm} (nm)	$D_{vesicle}$ (nm)	$T_{membrace}$ (nm)	N_{agg}
$PLMA_{22}$-P(0.9MMA-*stat*-0.1LMA)$_{47}$	Spheres	18.9 ± 1.9	-	-	-	-	201
$PLMA_{22}$-P(0.9MMA-*stat*-0.1LMA)$_{113}$	(Branched) Worms	-	20.0 ± 2.4	120	-	-	337
$PLMA_{22}$-P(0.9MMA-*stat*-0.1LMA)$_{247}$	Vesicles	-	-	-	172 ± 126	15.4 ± 1.6	25,600
$PLMA_{41}$-P(0.9MMA-*stat*-0.1LMA)$_{28}$	Spheres	19.8 ± 1.1	-	-	-	-	139
$PLMA_{41}$-P(0.9MMA-*stat*-0.1LMA)$_{282}$	Spheres	43.0 ± 4.5	-	-	-	-	513

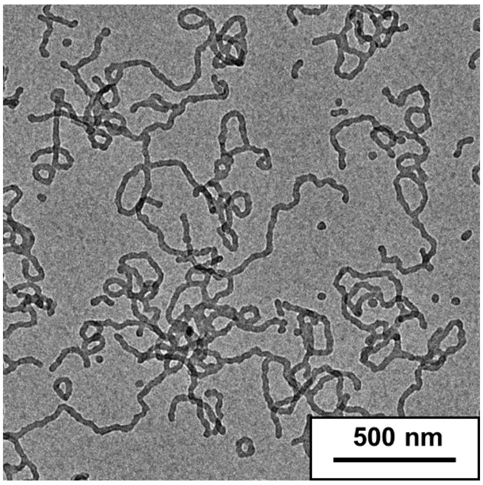

Figure S5. Representative TEM image obtained for the $PLMA_{22}$-P(0.9MMA-*stat*-0.1LMA)$_{228}$ worms that are formed after heating a 0.1% w/w dispersion of $PLMA_{22}$-P(0.9MMA-*stat*-0.1LMA)$_{228}$ vesicles (originally prepared in mineral oil but then diluted with *n*-dodecane) to 140 °C.

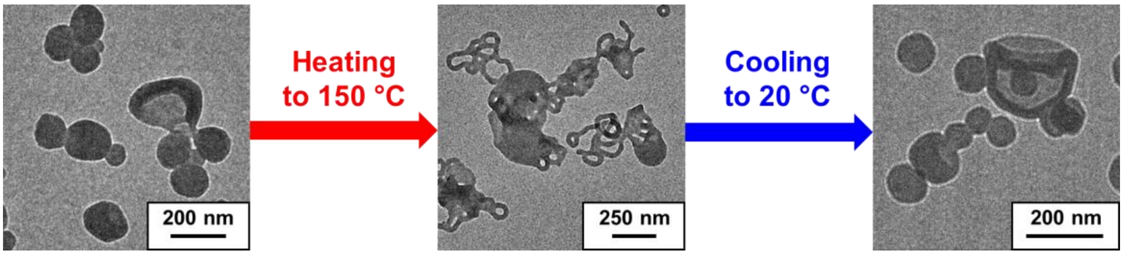

Figure S6. Representative TEM images recorded for the morphology transition exhibited by PLMA$_{22}$-P(0.9MMA-*stat*-0.1LMA)$_{228}$ vesicles prepared at 20% w/w solids in mineral oil. The initial copolymer dispersion was heated to 150 °C and equilibrated for 30 min at this temperature, prior to dilution with hot *n*-dodecane, cooling from 150 °C to 20 °C and ageing for 24 h at 20 °C.

Table S4. Summary of DLS data obtained for thermoresponsive PLMA$_{22}$-P(0.9MMA-*stat*-0.1LMA)$_{113}$ worms and PLMA$_{22}$-P(0.9MMA-*stat*-0.1LMA)$_{228}$ vesicles prepared at 20% w/w solids in mineral oil at 115 °C. The 20% w/w worm dispersion was heated to 150 °C, equilibrated for 1 h, then cooled to 20 °C and stored at this temperature for 24 h before DLS analysis. The vesicle dispersion was treated similarly, except that it was heated to 170 °C rather than 150 °C.

**Target Copolymer Composition**	**Experimental Conditions**	**DLS**		**TEM Morphology**
		**D_h (nm)**	**PDI**	
PLMA$_{22}$-P(0.9MMA-*stat*-0.1LMA)$_{113}$	Original	229	0.45	Worms
PLMA$_{22}$-P(0.9MMA-*stat*-0.1LMA)$_{113}$	Heated to 150 °C	35	0.04	Spheres
PLMA$_{22}$-P(0.9MMA-*stat*-0.1LMA)$_{113}$	Cooled to 20 °C	166	0.32	Worms
PLMA$_{22}$-P(0.9MMA-*stat*-0.1LMA)$_{228}$	Original	140	0.12	Vesicles
PLMA$_{22}$-P(0.9MMA-*stat*-0.1LMA)$_{228}$	Heated to 150 °C	104	0.16	Worms
PLMA$_{22}$-P(0.9MMA-*stat*-0.1LMA)$_{228}$	Cooled to 20 °C	129	0.09	Vesicles

References

(1) György, C.; Verity, C.; Neal, T. J.; Rymaruk, M. J.; Cornel, E. J.; Smith, T.; Growney, D. J.; Armes, S. P. RAFT Dispersion Polymerization of Methyl Methacrylate in Mineral Oil: High Glass Transition Temperature of the Core-Forming Block Constrains the Evolution of Copolymer Morphology. *Macromolecules* **2021**, *54*, 9496–9509.

(2) Fielding, L. A.; Lane, J. A.; Derry, M. J.; Mykhaylyk, O. O.; Armes, S. P. Thermo-Responsive Diblock Copolymer Worm Gels in Non-Polar Solvents. *J. Am. Chem. Soc.* **2014**, *136*, 5790–5798.

(3) Trent, J. S. Ruthenium Tetraoxide Staining of Polymers: New Preparative Methods for Electron Microscopy. *Macromolecules* **1984**, *17*, 2930–2931.

(4) Pauw, B. R.; Smith, A. J.; Snow, T.; Terrill, N. J.; Thünemann, A. F. The Modular Small-Angle X-Ray Scattering Data Correction Sequence. *J. Appl. Crystallogr.* **2017**, *50*, 1800–1811.

(5) Ilavsky, J.; Jemian, P. R. Irena: Tool Suite for Modeling and Analysis of Small-Angle Scattering. *J. Appl. Crystallogr.* **2009**, *42*, 347–353.

(6) Pedersen, J. S. Form Factors of Block Copolymer Micelles with Spherical, Ellipsoidal and Cylindrical Cores. *J. Appl. Crystallogr.* **2000**, *33*, 637–640.

(7) Bang, J.; Jain, S.; Li, Z.; Lodge, T. P.; Pedersen, J. S.; Kesselman, E.; Talmon, Y. Sphere, Cylinder, and Vesicle Nanoaggregates in Poly(Styrene-b-Isoprene) Diblock Copolymer Solutions. *Macromolecules* **2006**, *39*, 1199–1208.
